# N-acetyltransferase 10 promotes cutaneous wound repair via the NF-κB-IL-6 axis

**DOI:** 10.1038/s41420-023-01628-2

**Published:** 2023-08-29

**Authors:** Ben Wang, Jin Zhang, Guo Li, Chenzhong Xu, Langmei Yang, Jie Zhang, Yalan Wu, Ye Liu, Zuojun Liu, Ming Wang, Ji Li, Xiaolong Tang, Baohua Liu

**Affiliations:** 1 Department of Dermatology, Hunan Key Laboratory of Aging Biology, National Clinical Research Center for Geriatric Disorders, Xiangya Hospital, Central South University, Changsha, China; 2 Shenzhen Key Laboratory for Systemic Aging and Intervention (SKL-SAI), National Engineering Research Center for Biotechnology (Shenzhen), International Cancer Center, Guangdong Key Laboratory of Genome Stability and Human Disease Prevention, Guangdong Provincial Key Laboratory of Regional Immunity and Diseases, Department of Biochemistry & Molecular Biology, School of Basic Medical Sciences, Shenzhen University, Shenzhen, China; 3 Department of Histology and Embryology, School of Basic Medical Sciences, Central South University, Changsha, China; 4 School of Biomedical Sciences, Hunan University, Changsha, China

**Keywords:** Cell growth, Trauma

## Abstract

Cutaneous wound healing, an integral part for protection of skin barrier, is a complex biological process and intimately associated with keratinocyte migration. However, mechanisms regulating keratinocyte migration in the process of cutaneous wound repair remain largely unknown. Here, we found that N-acetyltransferase 10 (NAT10) is essential for cutaneous wound repair in an in vivo skin wound healing model—a significant delay of wound repair in *Nat10* haploinsufficient mice and a remarkable inhibition of keratinocyte migration by *NAT10* knockdown in an in vitro keratinocyte migration model. We further demonstrate that loss of *NAT10* expression attenuates the wound-induced IL-6/IL-8 expression through inhibiting NF-κB/p65 activity in keratinocytes. By deeply digging, silencing *NAT10* compromises the level of nuclear p65 by facilitating its poly-ubiquitination, thus accelerates its degradation in the nucleus. Notably, we detected a strong positive correlation between the expression of NAT10 and relevant NF-kB/p65-IL6 signaling activity in mouse wound skin tissues. Overall, our study reveals an important role of NAT10 on cutaneous wound repair by potentiating NF-κB/p65-IL-6/8-STAT3 signaling. Targeting NAT10 might be a potential strategy for the treatment of skin wound dysfunctions and related diseases.

## Introduction

One main function of the skin is to protect internal environment and homeostasis of the body from the invasion of harmful elements in external environment, known as skin barrier [1]. When disrupted by external injury or some certain diseases and in order to rebuild the integrity of skin barrier, skin initiates cutaneous wound healing process, i.e., wound repair, to ensure immediate response and quick recovery [2]. Skin repair disorders may lead to chronic unhealed skin ulcer that seriously affects the life quality, and cause severe defects of other organs and even death under some circumstances [3]. Cutaneous wound healing is composed of several delicately organized procedures. Firstly, tissue injury disrupts blood vessels and extravasation of blood constituents disturbs the regional hemostasis; then, keratinocytes, fibroblasts, and infiltrating inflammatory cells at the site of injury initiate the inflammatory response to remove harmful substances; lastly, accompanying with degradation of extracellular matrix and migration and proliferation of epidermal cells, tissue remodeling starts, and wound healing is thoroughly finished [4]. Notably, keratinocytes basically run through the whole process of skin repair by releasing inflammatory cytokines, self-migrate, and secreting growth factors, which promote proliferation of other skin cells [5]. However, the underlying mechanisms that regulate the function of keratinocyte during skin repair are not well explored.

Acetylation is one of the most commonly discovered post-translation modifications for amounts of proteins, which plays important roles in various physiological and pathological progressions, as well as skin barrier protection [6]. Acetylation-related regulators, for example, acetylases and deacetylases, are involved in the cutaneous wound repair [6]. N-acetyltransferase 10 (NAT10), a member of the general control non-repressible 5 (GCN5)-related N-acetyltransferase (GNAT) family, is the only one so far identified with both acetylase domain and RNA binding domain. Recently, with dozens of novel substrates being discovered, NAT10 is sparkling because of its involvement in multiple biological or pathological processes [7]. For instances, NAT10 directly acetylates MORC2 to sensitize DNA-damaging, which potentiates the efficacy of chemotherapy and radiotherapy in breast cancer [8]; NAT10 is reported as a cellular stress sensor which acetylates p53 and counteracts Mdm2 action, thus eliciting p53 activation under DNA damage-related stress [9]; NAT10 interacts with Che-1 in the anabolism-catabolism transition in response to energy stress [10]. Moreover, inhibiting NAT10 extended the healthy lifespan in an *Lmna*^G609G^ progeria mouse model, suggesting its intimate link with aging [11]. Recent findings revealed that N^4^-acetylcytidine (ac4C) modification of tRNA, rRNA, and mRNA is catalyzed by NAT10, improving the stability of RNA and protein translation efficiency [12, 13]. For example, NAT10 has been found to execute ac4C acetylation for mRNA of RUNX2 and COL5A1, which prevents ovariectomy-induced bone loss and promotes gastric cancer metastasis, respectively [14, 15]. Albeit current findings dictate crucial role of NAT10 in cell apoptosis, proliferation, and motility, thus modulating cancer progression, metabolism and aging, whether NAT10 regulates cutaneous wound healing and skin barrier maintaining is largely unknown. In this study, we investigated the potential function of NAT10 in wound repair by using keratinocyte as an in vitro cell model and scratching-wound as an in vivo mouse model. Our results demonstrate that NAT10 is critical for potentiating NF-κB/p65-IL-6 signaling which ensures efficient skin repair, and *NAT10* haploinsufficiency elicits apparent delayed skin repair. Our findings highlight NAT10 as a potential target that might be useful for the treatment of disorders associated with skin repair dysfunction.

## Results

### *Nat10* haploinsufficiency delays wound repair in mice

NAT10 regulates multiple physiological and pathological processes [7]. However, the roles of NAT10 in skin barrier, a delicately manipulated procedure, are barely delineated. Here, using a canonical wound healing model, we investigated the functions of NAT10 in skin repair. We generated a *Nat10* knockout (KO) allele by using the standard CRISPR-Cas9 methodology (Fig. S1A, B in Supporting Information). After backcross, we successfully obtained heterozygous mice (Fig. S1C in Supporting Information). Notably, consistent with the previous finding [11], the *Nat10* KO mice were also embryo lethal, while no apparent difference was observed between the wildtype (WT) and *Nat10*^+/−^ littermates, which displayed comparable body size and skin morphogenesis (Fig. S1D in Supporting Information).

**Fig. 1:**
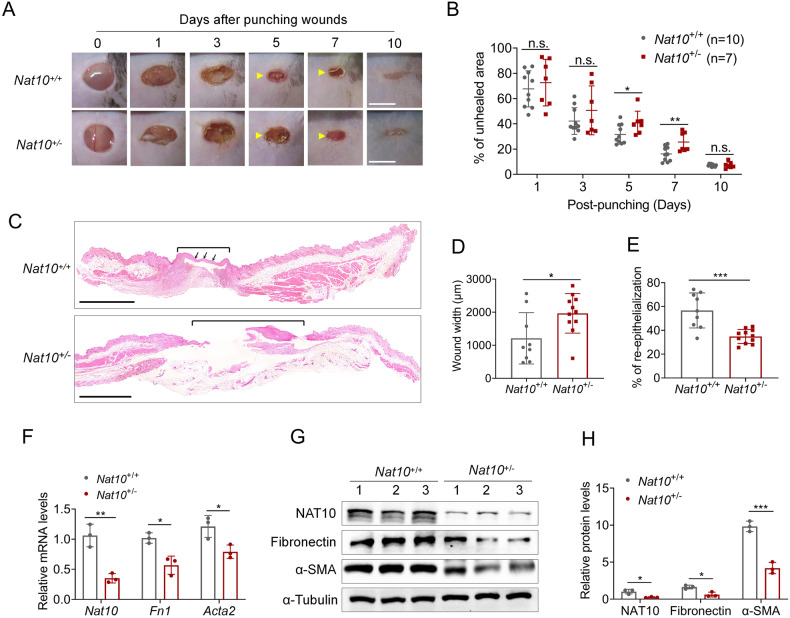
*Nat10* haploinsufficiency delays wound repair. **A** Representative images showing the wound healing process of *Nat10*^+/−^ mice (*n* = 7) and their WT littermates (*n* = 10). Yellow arrows indicate the evident delayed wound repair in *Nat10*^+/−^ mice. Scale bars, 5 mm. **B** Wound closure of (**A**) was quantified and unhealed rate was presented as the percentage of wound compared with the initial wound area size. n.s., no significance. **C** H&E staining of the wounded skin tissues derived from WT and *Nat10*^+/−^ mice on day 5 after punching. Black lines, unhealed wound; Arrows, fresh skin with re-epithelialization. Scale bars, 100 μm. **D** The chart showing quantified wound width basing on the H&E staining images (WT: *n* = 9, *Nat10*^+/−^: *n* = 11). **E** Quantification of the re-epithelialization in wounds of both genotypes based on the H&E staining images (WT: *n* = 9, *Nat10*^+/−^: *n* = 11). **F** qPCR analysis of *Nat10*, *Fn1* and *Acta2* expression in wounded skin tissues collected from WT and *Nat10*^*+/−*^ mice, n = 3 for each genotype. Immunoblot analysis of NAT10, FN1 and α-SMA protein expression in wounded skin tissues of WT and *Nat10*^+/−^ mice at day 5 after wounding (**G**), and the relative quantification were presented in (**H**) using ImageJ Plus 6.0 (*n* = 3 mice for each group). The data are presented as the means ± SEM, * *P* < 0.05, ** *P* < 0.01, *** *P* < 0.001, determined by Student’s *t* test.

To evaluate whether *Nat10* affects wound healing process, a full-thickness 6-mm punch-biopsy wound was made, basing on the previously reported method [16], in the dorsal skins of WT and *Nat10*^+/−^ mice (Fig. 1A, day 0) and then the wound morphology was recorded at day 0, 1, 3, 5, 7, and 10 after punch. As shown, the wound healing speed of WT (*n* = 10) and *Nat*10^+/−^ mice (*n* = 7) displayed no significant difference at early stage (on day 1 and day 3). However, apparent delayed repair rate was obvious in late period (on day 5 and day 7) in *Nat*10^+/−^ mice compared with WT littermates (Fig. 1A). Quantification of unhealed skin area affirmed slower repair speed in *Nat*10^+/−^ mice (Fig. 1B). We next performed histologic analysis by H&E staining of skin biopsies collected at day 5 when apparent morphology difference was first observed. As shown, the average wound diameter in *Nat10*^+/−^ mice was longer than WT littermates (Fig. 1C, D). Re-epithelialization is a hall marker of successful wound repair [17]. Notably, the wound edges of *Nat10*^+/−^ mice had less extent of re-epithelialization (indicated by arrows) compared with WT mice (Fig. 1C, F), further consolidating the finding of delayed wound healing in *Nat10*^+/−^ mice. To complete wound repair, fibrosis is indispensable and considered to play irreplaceable roles because reconstituting fresh skin needs amount of various matrix proteins [18]. Indeed, significant lower expression of fibronectin and α-SMA, in both mRNA and protein levels, was observed in skin samples of wound area derived from *Nat10*^+/−^ mice when compared with WT mice (Fig. 1F–H), suggesting an accompanied delay of fibrosis. Taken together, these data demonstrate that NAT10 haploinsufficiency in mice compromises wound repair.

### NAT10 knockdown inhibits keratinocyte migration

**Fig. 2:**
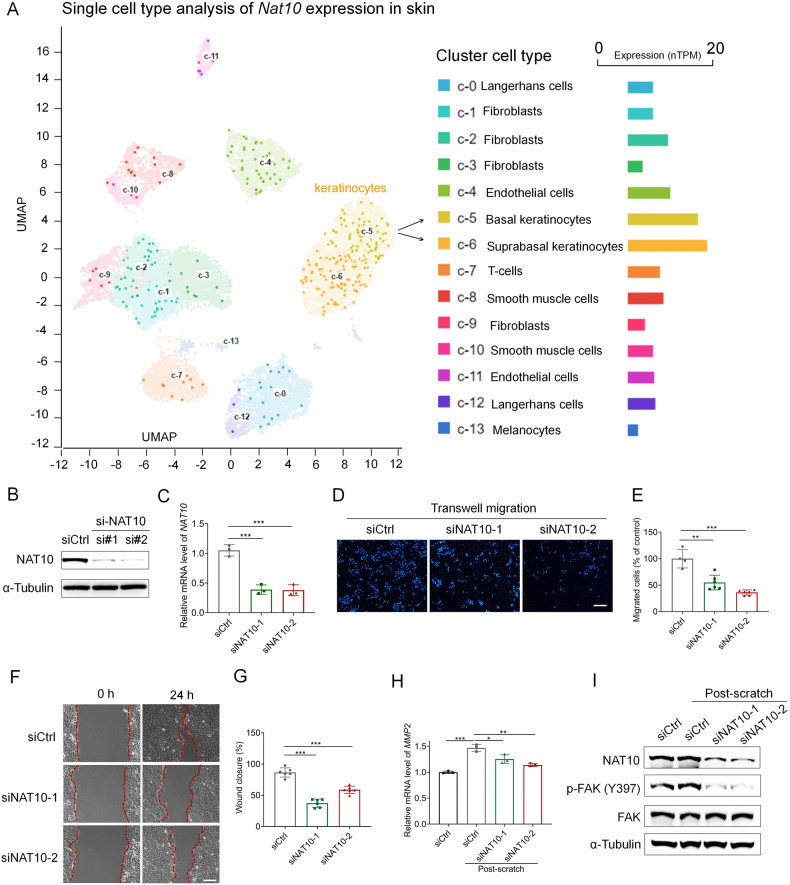
NAT10 is crucial for keratinocyte migration. **A** Single cell RNA-sequencing analysis showing the expression of *Nat10* in various skin cell types. Original data were from public online tool of The Human Protein Atlas. **B**, **C** Immunoblot and qPCR analysis showing NAT10 protein and mRNA levels in HaCaT cells with or without knocking down NAT10 expression after 72 h of transfection (*n* = 3 for each group). Representative images (**D**) and related quantification (**E**) showing the migrated cells with or without NAT10 knockdown by using trans-well assay for 24 h. Scale bars, 50 μm. **F**, **G** Representative images (**F**) and related quantitative analysis showing the wound closure of HaCaT cells treated with or without NAT10 siRNA by using wound healing assays for 24 h (*n* = 6 for each group). Scale bars, 50 μm. **H** qPCR analysis of *MMP2* expression in HaCaT cells with indicated treatments after scratching for two hours (*n* = 3 for each group). **I** Immunoblotting analysis of p-FAK (Y397) in HaCaT cells with or without NAT10 knockdown after scratching. Data are presented as the means ± SEM. *P < 0.05, **P < 0.01, ***P < 0.001, obtained by Student’s *t* test.

We next asked how NAT10 affected skin repair. Interestingly, based on the analysis of single cell RNA-sequencing data of skin tissue from a public online tool (The Human Protein Atlas), we found a much higher expression of NAT10 in keratinocyte cells, when comparing with other skin cell types, for example, monocytes and fibroblast cells (Fig. 2A). Indeed, once wound injury is created, skin keratinocyte is one of the earliest activated cell types and its migration is crucial to initiate the following wound repair [5]. Those findings encouraged us to explore the roles of NAT10 in keratinocyte. To that end, *NAT10* expression was silenced in a widely-used keratinocyte cell line, i.e., HaCaT cells [19], by RNA interference (RNAi). As expected, both mRNA and protein levels of NAT10 were ablated (Fig. 2B, C). We then set out to analyze the effect of *NAT10* silence on keratinocyte migration. As evaluated by trans-well migration assay and scratching-induced wound healing assay, it was clear that downregulation of NAT10 expression in HaCaT cells prominently inhibited cell migration (Fig. 2D, E) and resulted in apparent delay of wound closure (Fig. 2F, G). Meanwhile, *NAT10* knockdown elicited little change on cell proliferation and viability when compared with scramble controls (Fig. S2A, B in Supporting Information), emphasizing the major contribution of NAT10 for keratinocyte migration. Moreover, silencing *NAT10* impaired wound-induced up-regulation of MMP-2 which is critical for keratinocyte collective migration during wound healing [20] (Fig. 2H). Moreover, the activity of FAK, indicated by the phosphorylation of Y397 and the up-stream regulator of MMP2, was significantly downregulated in *NAT10* knockdown cells (Fig. 2I). Taken together, these data suggest that NAT10 modulates wound healing process, at least partially, through potentiating keratinocyte migration.

### Silencing NAT10 attenuates the wound-induced IL-6/IL-8 expression

**Fig. 3:**
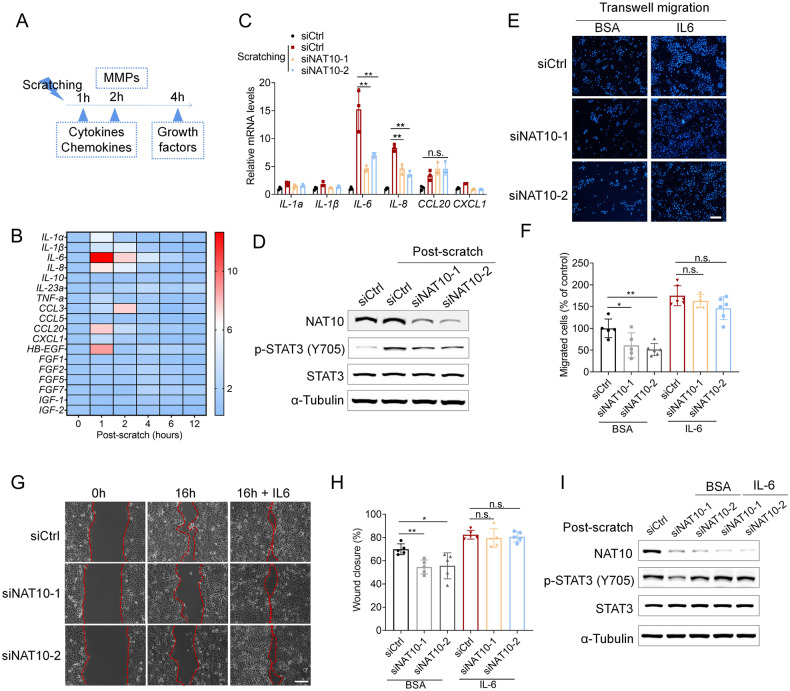
NAT10 knockdown inhibits keratinocyte migration through impairing IL-6 expression. **A** The schematic diagram described the induction of cytokines, chemokines, MMPs and growth factors expression by scratch-induced wound at different time points. **B** The heat map showing the production of cytokines, chemokines, MMPs and growth factors at serial time points in keratinocyte cells post scratching. **C** qPCR analysis of the expression of several key cytokines in HaCaT cells treated with control or NAT10 siRNAs in the scratch-wounded model (*n* = 3 for each group). n.s., no significance. **D** Immunoblotting analysis showing level of phosphorylated STAT3 in HaCaT cells treated with control or NAT10 siRNAs in the wound healing model. **E**, **F** Representative images (**E**) and related quantitative analysis showing the trans-well assays (lasting for 24 h) of HaCaT cells with or without NAT10 knockdown in the presence of absence of rIL-6. rIL-6, exogenous recombinant human IL-6 (*n* = 5 for each group). Scale bars, 50 μm. **G**, **H** Representative images and related quantitative analysis showing the in vitro wound healing assay of HaCaT cells (lasting for 16 h) treated with NAT10 siRNA or rIL-6 (*n* = 3 for each group). Scale bars, 50 μm. **I** Immunoblot analysis of phosphorylated STAT3 expression in HaCaT cells treated with NAT10 siRNAs and rIL-6 or not. Data are represented as mean ± SEM. * *P* < 0.05, ** *P* < 0.01, determined by Student’s *t* test.

We found that both mRNA and protein levels of NAT10 were merely affected in HaCaT cells upon scratch-induced wound, implicating that NAT10 regulating keratinocyte migration is not due to gain-of-expression but possibly imposed by an intrinsic mechanism (Fig. S3A, B in Supporting Information). We further asked how NAT10 affected keratinocyte cell migration-related regulators, for example as wound-induced cytokines, chemokines, matrix metalloproteinases (MMPs) and growth factors released by keratinocytes that are critical for wound-healing process [2]. Interestingly, wound conferred cells expressing cytokines and chemokines mainly peaked at 1–2 h after scratching, MMPs at 2 h, and growth factors at 4 h (Fig. 3A, B). Indeed, those early secreted cytokines are recognized as key triggers for wound repair initiation, such as IL6 [21]. Of note, NAT10 silencing resulted in more apparent decrease of wound-induced IL-6 and IL-8 expression compared with other cytokines (Fig. 3C). Accordingly, wound-induced STAT3 activation (p-STAT3), which is considered as one of the major effectors of IL6 or IL8 signaling [22], was significantly declined by *NAT10* knockdown (Fig. 3D). We thus assumed that *NAT10* knockdown might suppress keratinocyte migration through downregulation of IL6/IL8-STAT3 activity. In terms of STAT3 activation, IL6 and IL8 possess similar effect [23], we thus used recombinant human IL-6 (rIL6) to perform the rescue experiment. We found that supplementing rIL6 largely restored the ability of cell migration, as assessed by both trans-well and wound-healing assays (Fig. 3E-H). Likewise, rIL6 almost completely recovered the level of p-STAT3 and MMP2 in *NAT10* knockdown HaCaT cells (Fig. 3I and Fig. S4 in Supporting Information). Together, these results demonstrate that NAT10 is crucial for keratinocyte cell migration through promoting wound-induced IL-6 expression.

### NF-κB/p65 underlies NAT10-mediated IL6 regulation during wound repair

**Fig. 4:**
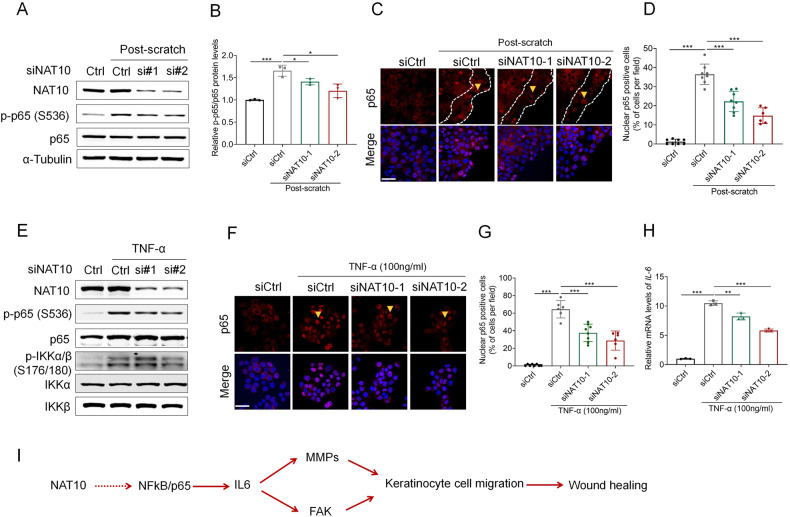
NF-κB/p65 underlies NAT10-mediated IL6 regulation during wound repair. **A**, **B** Immunoblot (**A**) analysis of p65 activation in scratch-wounded keratinocyte HaCaT cells treated with control or NAT10 siRNAs. Cells lysates were prepared after scratch for 1 h. Relative levels of p-p65/p65 protein were quantified by ImageJ Plus 7.0 in (B) (*n* = 3 for each group). **C**, **D** Representative images (**C**) showing immunofluorescence staining of p65 in keratinocyte HaCaT cells treated with control or NAT10 siRNAs post scratch for 1 h. Percentage of nuclear p65 positive cells were calculated (**D**) (*n* = 8 for each group). Dot line indicating cells along the wounded edge. Scale bars, 50 μm. Immunoblotting (**E**) showing p65 activation in HaCaT cells treated with or without NAT10 siRNAs and addition of TNFα for 15 min. **F**, **G** Representative images showing immunofluorescence staining of p65 in HaCaT cells treated with or without NAT10 siRNAs and addition of TNFα for 15 min (**F**).The percentage of nuclear p65-positive cells were calculated (**D**, *n* = 6 for each group). Scale bars, 50 μm. **H** qPCR analysis of *IL6* expression in HaCaT cells with or without treatment of TNFα (lasting for 1 h) and NAT10 siRNAs (*n* = 3 for each group). **I** Schematic diagram illustrating the mechanism of NAT10 affecting keratinocyte migration through NF-kB/p65-IL6 signaling. Data are represented as mean ± SEM. * *P* < 0.05, ** *P* < 0.01, *** *P* < 0.001, determined by Student’s *t* *t*est.

IL-6/IL8 expression is mainly regulated by NF-κB/p65 in various cellular conditions [24, 25]. Moreover, previous studies have demonstrated the activation of NF-κB/P65 during wound-healing [26, 27]. We thus asked whether NAT10 regulates IL6 expression via NF-κB/p65 pathway. Indeed, p65 activity (p-p65) was prominently increased after scratching wound (Ctrl group, Fig. 4A). Silencing *NAT10* remarkably attenuated such activation (Fig. 4A, B). To fulfill the function in transcription regulation, phosphorylated p65 translocates into nucleus [28]. Consistently, determined by immunofluorescence staining, we found that the intensity of nuclear p65 was significantly lower in HaCaT cells with silenced NAT10, and the difference was more evident in cells along the wound edge (Fig. 4C, D). To gain more evidence of NAT10 regulating NF-κB/p65 activation, we employed TNF-α, a widely-used stimulator of NF-κB pathway, to assess whether similar effect could be recapitulated. As shown, NAT10 silencing suppressed TNF-α-induced p65 phosphorylation (Fig. 4E), and nuclear accumulation (Fig. 4F, G). If NAT10 regulating IL6 is governed by NF-κB/p65, TNF-α-stimulated IL6 expression would be affected by NAT10 manipulation. Indeed, TNF-α-induced IL6 expression was greatly attenuated in *NAT10* silenced HaCaT cells (Fig. 4H). Together, these data implicate a NAT10-NF-κB/p65-IL6 signaling axis in regulating wound repair (Fig. 4I).

### Silencing *NAT10* compromises nuclear p65 stability via facilitating its poly-ubiquitination

**Fig. 5:**
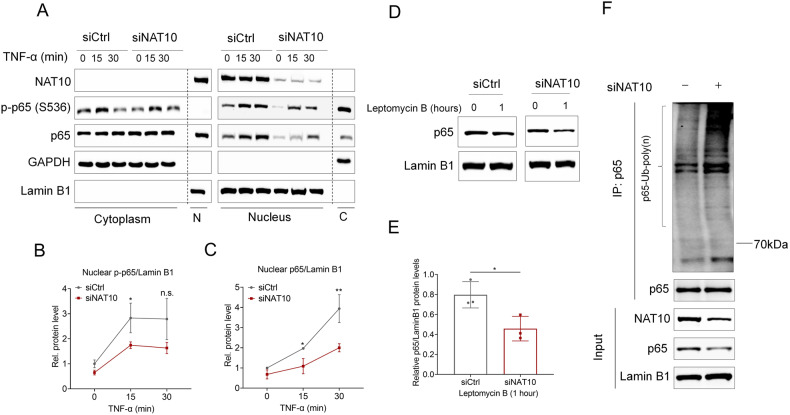
NAT10 maintains nuclear p65 stability via restraining its poly-ubiquitination. **A** Immunoblotting analysis of cytoplasmic and nuclear p65 activation in HaCaT cells with or without NAT10 siRNAs and TNFα treatment. GAPDH and Lamin B1 marked the cytoplasmic and nuclear protein fraction, respectively. N, nucleus; C, cytoplasm. **B**, **C** The relative levels of nuclear p65/Lamin B1 and p-p65/Lamin B1 in (**A**) were quantified from 3 independent experiments. **D**, **E** Immunoblotting (**D**) showing nuclear p65 levels in Leptomycin B-treated HaCaT cells with control or NAT10 siRNA treatment. The relative level of p65/Lamin B1 was quantified in (**E**) (*n* = 3 for each group). **F** Immunoblotting showing the poly-ubiquitination of nuclear p65 in HaCaT cells with or without NAT10 knockdown. Data are presented as the means ± SEM. * *P* < 0.05, ** *P* < 0.01, determined by Student’s *t* test.

One of the key regards to NF-κB/p65 activation is associated with relevant receptor of TNF-α [29]. However, we found little change of TNFR1 or TNFR2 expression level in HaCaT cells upon *NAT10* interference (Fig. S5 in Supporting Information), thus excluding defect in receptor-mediated signal initiation. NF-κB/p65 activation is tightly regulated by the cytoplasm-to-nucleus translocation. We thus asked whether NAT10 mediates this process. To that end, nuclear and cytoplasmic fractions derived from HaCaT cells with or without *NAT10* knockdown and TNFα were subjected to immunoblotting. Interestingly, p-p65 and total p65 in the nucleus fraction showed a very apparent change, displaying more extent of decrease by *NAT10* knockdown (Fig. 5A–C), while the cytoplasmic portion had less change (Fig. 5A, and Fig. S6A, B in Supporting Information), indicating that NAT10 mainly regulates nuclear p65. This finding seems plausible as NAT10 is a nucleus-resided protein [30]. Additionally, applying phosphatase inhibitor LB-100, the inhibitor of PP2A which dephosphorylates p65 [31, 32], did not markedly affect the p65 nuclear distribution in HaCaT cells with *NAT10* knockdown or not (Fig. S6C in Supporting Information), further excluding the possibility of PP2A-mediated regulation of nuclear p65. We next asked whether NAT10 affected p65 nuclear exportation, which is mainly manipulated by nucleocapsid CRM1 [33]. However, after treatment with Leptomycin B (LMB), the CRM1 inhibitor [34] that restrains nuclear p65 exportation, we found that p65 level was yet not apparently restored in *NAT10* silenced cells, suggesting the decrease of nuclear p65 by *NAT10* knockdown was not due to the change of nuclear p65 exportation (Fig. 5D, E). It should be noted that, on one hand, LMB treatment restrained the export of nuclear p65, on the other hand, cytoplasmic p65 continually translocated into nucleus. Considering the combined effects, we reasoned that p65 nuclear proportion should be underwent much faster turnover upon *NAT10* knockdown. It has been revealed the degradation of nuclear p65 is mainly mediated by ubiquitination-mediated proteasome pathway [35, 36]. We thus determined whether the ubiquitination of p65 was affected by NAT10. Indeed, *NAT10* knockdown significantly enhanced p65 poly-ubiquitination in the nucleus, indicating that the low level of nuclear p65 is, at least partially, owing to faster degradation (Fig. 5F). Collectively, our results suggest that NAT10 maintains the steady activity of nuclear p65 through restraining its poly-ubiquitination-mediated degradation in nucleus.

### NF-kB/p65-IL6 signaling is positively correlated with NAT10 during wound repair

**Fig. 6:**
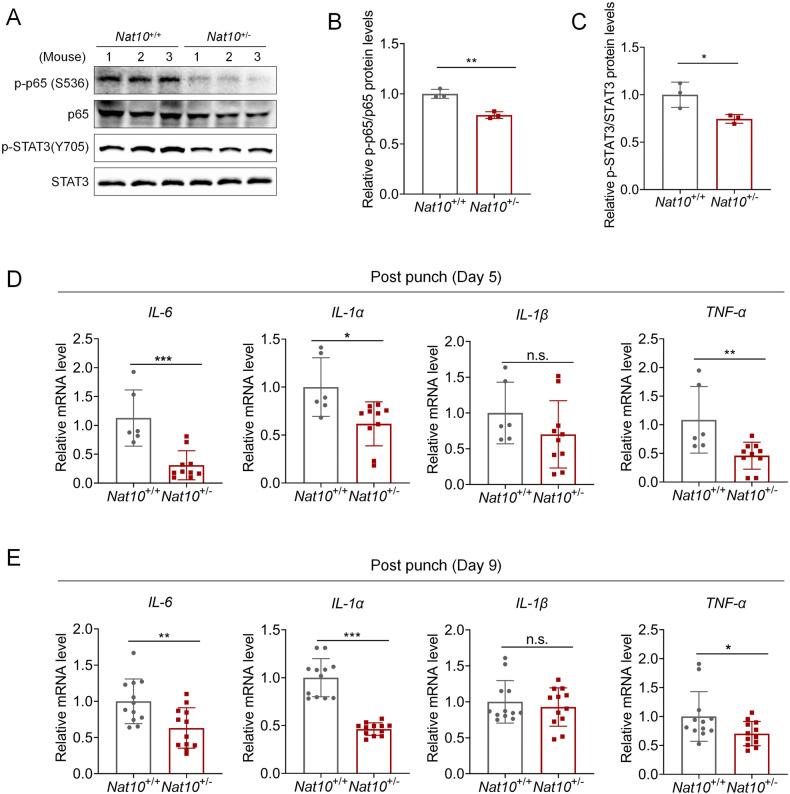
Positive correlations of NAT10 expression and NF-kB/p65-IL6 signaling in wound skin tissues. **A**–**C** Immunoblotting (**A**) showing p-p65 and p-STAT3 expression level derived from wounded skin tissues of WT and *Nat10*^+/−^ mice (*n* = 3 mice for each group). The indicated quantification was displayed in (**B**) and (**C**). **D**, **E** qPCR analysis of *IL-6*, *IL-1α*, *IL-1β* and *TNFα* levels derived from wounded skin tissues of WT and *Nat*10^+/−^ mice at day 5 (WT: *n* = 6; Nat10^+/−^: *n* = 10) and day 9 (WT: *n* = 12; Nat10^+/−^: *n* = 12) after punching. Data are presented as the means ± SEM. *P < 0.05, **P < 0.01, and ***P < 0.001, determined by Student’s *t* test.

We next examined the in vivo correlation of NAT10 and NF-kB/p65-IL6 signaling. The levels of p-p65 and p-STAT3 were significantly decreased in wound skin tissues of *Nat10*^+/−^ mice compared with those of WT mice, detected at day 5 after punching (Fig. 6A-C; Fig. S7A, B). In addition, a greater decline of cytokines that are in the control of NF-kB/p65 signaling, i.e., IL-6, IL-1α and TNF-α, was consistently observed in skin tissues of *Nat10*^+/−^ mice (Fig. 6D). Such changes remained even to day 9, when skin repair was almost completed in both genotypes (Fig. 6E).

## Discussion

To maintain skin barrier homeostasis which is frequently disrupted by skin wound, skin autonomously activates the cutaneous repair process. Keratinocyte, as one of the important components of the skin, is a determinant factor for adequate and precise wound healing, which requires keratinocyte migrating into the right place at the right time. Dysfunction or impaired keratinocyte migration causes delayed wound healing, which may lead to chronic unhealed skin ulcer, defects of other organs and even death. In our study, we identified NAT10 as a key regulator of keratinocyte migration. Downregulation of NAT10 apparently attenuates wound-induced IL6/IL8 expression, compromises keratinocyte cell migration, and thus significantly delays mouse skin repair in both in vivo and in vitro models. Mechanically, NAT10 potentiates the NF-κB/p65 signaling through restraining nuclear p65 poly-ubiquitination. Our findings highlight NAT10 as a critical regulator of wound repair and skin barrier homeostasis.

Protein acetylation regulates keratinocyte migration during wound healing. It has been shown that the impairment of acetylase P300/CBP-associated factor (PCAF) delays skin repair, and the PCAF activator pentadecylidenemalonate 1b (SPV-106) promotes wound repair [6]. Treatment with Histone deacetylase inhibitor elicits increased wound closure through enrichment of specific subset of macrophages [37]. The sirtuin family, NAD^+^-dependent and conserved longevity related deacetylases [38], also regulates wound healing. Sirt1 promotes corneal epithelial wound healing through the deacetylation of p53 and enhancing IGFBP3/IGF-1/AKT pathway [39]. NAT10 functions as an acetylase [9]. Our study, to some extent, further emphasizes the great importance of acetylation regulation implicates wound repair process.

NF-κB/p65 pathway is critical for keratinocyte migration, which is activated by phosphorylation of p65 and subsequent translocation to the nucleus to drive the expression of wound healing related genes, such as IL6/IL8. One mechanism leading p65 inactivation is ubiquitination-mediated degradation [35, 36, 40]. For instance, an elegant study has unraveled that after p65 entering the nucleus, PDLIM2, as a nuclear ubiquitin E3 ligase, binds to p65 and promotes its polyubiquitination. In current study, we unravel a novel regulator that NAT10 contributes for the stability of nuclear p65 and thus sustains long-term activation. The absence of NAT10 facilitates the degradation of nuclear p65 via poly-ubiquitination. Whether such effect is dependent on NAT10 acetylase activity to p65 or other targets merits further investigation. Indeed, we failed to detect interaction between p65 and NAT10, suggesting a less possibility of NAT10 directly acetylating p65. We assume an existence of some indirect mechanisms, for instances, through SIRT1 and SIRT7, two NAD-dependent deacetylases which are reported to form complex with NAT10 [10, 13], by which NAT10 might affect the activity of nuclear p65. As previously reported, SIRT1 S-nitrosylation-dependent acetylation of p65 contributes to muscle wasting in burn wound repair; and SIRT7 regulating p65 is involved in inflammation process, and TGF-β signaling transduction in the tissue repair process [41, 42].

Due to embryo lethality by *Nat10* total KO in mice, we used the heterozygotes for most of the in vivo phenotypical analyses. While NAT10 haploinsufficiency does remarkably delay wound skin repair, which, to some extent, demonstrates our views, we realize that keratinocyte-specific KO of *Nat10* mice would be helpful to exclude the unexpected effects and further consolidate the conclusion. Moreover, the seemingly mild phenotype that *Nat10*^+/−^ mice only showed delayed speed rate of wound healing at day 5 and 7 and finally completed wound repair at day 10, could be also explained by half loss of *NAT10*. Supporting this notion, abrogation of NAT10 in HaCaT keratinocyte cells in vitro elicits great inhibition of keratinocyte migration without apparent effect on cell viability and proliferation. We also identify that NAT10 has higher expression level in skin keratinocytes by analysis of single cell RNA sequencing data profiles from skin tissue. The results confirm the roles of NAT10 in wound healing via regulating keratinocyte migration. Nevertheless, in future study, generating *Nat10* conditional KO mice to evaluate the keratinocyte specific function is warranted.

In summary, our study demonstrates that NAT10 ensures efficient skin wound repair by potentiating NF-κB/p65-IL-6/8-STAT3 signaling which promotes keratinocyte migration. Our findings highlight NAT10 as a potential target for the treatment of skin wound dysfunction and related diseases.

## Materials and methods

### Animals

*Nat10*^**+/−**^ C57BL6 mice were generated by using the standard CRISPR-Cas9 methodology, purchased from Cyagen Company (Suzhou, China). The *Nat10*^*+/−*^ mice were backcrossed for at least three generations to separate potential off-target deletions. To create skin wound healing, the fur was removed from the back of the mice and full-thickness excision wounds using a 6-mm biopsy punch were made on the dorsal skin. The general pictures of wounds were taken by Canon camera (Oita, Japan) and skin wound tissues were harvested at days 0, 1, 5, 9 post-punching. The wound area was calculated using ImageJ Software (NIH, Bethesda, MD), and the wound healing speed was measured as a ratio of the wound area at each time normalized by the area at day 0. To perform histological analysis, those collected tissues at 5 days post-punching were fixed and then stained with Hematoxylin-Eosin (H&E staining). Relevant images were captured by microscope (Olympus, IXplore, Japan) for further analysis. Above mentioned data collection were performed by researchers who were blinded to the group information. Each genotype of mice (at least 6 mice) was randomly grouped and the mice initially punched with irregular skin wounds would be excluded for further study. All mice were maintained under specific pathogen-free conditions and the procedures were performed in accordance with protocols approved by the Committee for Experimental Animal Research of Shenzhen University.

### Cell culture

HaCaT (immortalized human keratinocyte cell line) cells were purchased from Kepu Company (Shenzhen, China) and cultured in Dulbecco’s modified Eagle’s medium (DMEM, high glucose, Corning) supplemented with 10% Fetal Bovine Serum (FBS, Gibco) and penicillin-streptomycin antibiotics (Gibco) in a 5% CO2 incubator at 37 °C. Confluent Cells were passed by trypsin with 0.25% EDTA (Gibco) with a ratio of 1:4. Other reagents used for additional treatments were: Recombinant Human IL-6 (rhIL6) (Peprotech, 200-06), Recombinant Human TNF-α (rhTNF-α) (Peprotech, 300-01 A), Leptomycin B (Selleck, S7580), LB 1-00 (Selleck, S7537) and MG132 (Beyotime, S1748). For analysis from cell culture, we plated cells in a random distribution and randomly assigned them to experimental groups.

### Cell migration assays

For scratching-wound healing assay, HaCaT cells were plated on 6-well culture plates until they reached 80–90% confluence, and then cells were scratched using a 100-μl pipette tip after they were starved in serum-free medium for 24 h. Next, cells were washed three times with PBS and cultured in fresh DMEM with 2% FBS. The scratched areas were recorded after scratching at the different time point using the ZEISS ZEN microscopy (ZEISS, Germany). The percentage of unhealed area at each time point was calculated by normalizing with the wound area at zero by using ImageJ software. For trans-well assay, a suitable number of cells (5*10^4^ cells/ml) were seeded in the upper chamber (Corning, China) and the lower chamber was incubated by DMEM with 2% FBS. Migrated cells were fixed, stained by DAPI (Beyotime, China) and migrated cell number was counted after pictures were taken.

### Plasmid transfection and RNA interference (RNAi)

Gene expression by RNAi was performed by specific siRNA oligos (GenePharma, China) which was transfected by using Lipofectamine 3000 (Thermo, USA) according to the manufacturer’s instructions. The two sequences of NAT10 siRNAs were listed as follows: 5'-AUGGAACACUGAACAUAAATT-3' and 5'-GGCCAAAGCUGUCUUGAAATT-3'. Knockdown efficiency was validated by immunoblotting or real-time PCR after 48–72 h of transfection as indicated in figure legends, and all the NAT10 knockdown studies were performed within 72 h after transfection.

### Total RNA extraction and quantitative qPCR analysis

Total RNA was isolated in Trizol reagent (Takara, Japan) according to a standard method. Total mRNA was reversely transcripted into cDNA by 5 × Primescript RT Master Mix (Takara, Japan). Quantitative real-time PCR was performed with 2 × SYBR Green Mix (Takara, Japan) in Bio-Rad detection system. Primer sequences used in this research are provided in Supplementary Table 1 (Table S1).

### Protein extraction and immunoblotting

Protein samples derived from mouse skins and cells were extracted by RIPA lysis buffer containing phosphatases and proteases inhibitor cocktails (Roche, USA). Protein concentration was determined by BCA protein assay kit (Pierce, USA). For immunoblotting analysis, protein samples were subjected with SDS-polyacrylamide gel electrophoresis and proteins were transferred to PVDF membranes (Millipore). After being blocked, and incubated with primary antibodies and indicated HRP-coupled secondary antibody, membranes were visualized with ECL and images were captured using the Bio-Rad system. Band intensities were detected, normalized, and quantified with the Image Lab 5.0 software (Bio-Rad). The following antibodies were used in this research: NAT10 (Abcam, ab194297, 1:1000 dilution), STAT3 (CST, 30835, 1:1000 dilution), p-STAT3 (Abcam, ab76315, 1:1000 dilution), p-p65 (CST, 3033, 1:1000 dilution), p65 (CST, 8242, 1:1000 dilution), p-FAK(CST, 8556, 1:1000 dilution), FAK (Abcam, ab40794, 1:1000 dilution), Lamin B1 (Abcam, ab133741, 1:1000 dilution), poly-Ubiquitin (CST, 3936, 1:1000 dilution), Fibronectin(CST, 26836, 1:1000 dilution), α-SMA(Proteintech, 14395-1-AP, 1:1000 dilution), α-Tubulin (Beyotime, AT819, 1:5000 dilution), and GAPDH (Beyotime, AG019, 1:5000 dilution).

### Immunoprecipitation (IP)

Cell samples were prepared in IP lysis buffer (150 mM NaCl, 25 mM Tris-HCl (pH 7.9), 5 mM MgCl2, 10% glycerol, 0.2 mM EDTA, 0.1% NP-40) with protease inhibitors (Roche Complete). Cleared cell lysates were then incubated with indicated antibodies and its normal IgGs, and 15 μl Protein A/G agarose beads (Invitrogen, USA) at 4 °C for 4 h. The immunoprecipitates were washed, eluted in Laemmli loading buffer, and analyzed by immunoblotting.

### Statistical analysis

Sample size was chosen according to generally accepted number in the field and no particular statistical methods were used to predetermine it. Some of the data collection and analyzes were not performed blind due to obvious differences between groups but at least two observers performed the experiments and independently analyzed the data. The data were presented as means ± s.e.m. or means ± s.d. with indicated statements in the manuscript. Statistical analyses were performed by SPSS 22.0 (IBM Corp, Armonk, NY) or GraphPad Prism 8 (GraphPad Software, Inc, La Jolla, CA). Statistical differences were determined by Student’s *t* test. *P* < 0.05 was considered with statistical significance. All experiments were repeated at least three times and all sample sizes were shown in the figures legends.

### Supplementary information

Supporting information

Original Data File

## Data Availability

All data that support the findings of this study are available from the corresponding authors upon reasonable request.
