# Supplementary material for: N-acetyltransferase 10 promotes cutaneous wound repair via the NF-κB-IL-6 axis
Source: Cell Death Discov. 2023 Aug 29;9:324. doi: 10.1038/s41420-023-01628-2 (PMC10465497; doi:10.1038/s41420-023-01628-2)
Supplement: Supplementary file 1 — Supporting information [file 41420_2023_1628_MOESM1_ESM.pdf]

Supporting Information

Table S1. Oligonucleotide primers used for quantitative PCR in this study

Gene		human	mouse
**Gapdh**	Forward	CACCCACTCCTCCACCTTTGAC	AGGTCGGTGTGAACGGATTTG
	Reverse	GTCCACCACCCTGTTGCTGTAG	TGTAGACCATGTAGTTGAGGTCA
**Nat10**	Forward	ATAGCAGCCACAAACATTCGC	TCATTGAGAATGGCGTAGCTG
	Reverse	ACACACATGCCGAAGGTATTG	CACAGTTGCCTTGGACAACAT
**Fn1**	Forward		ATGTGGACCCCTCCTGATAGT
	Reverse		GCCCAGTGATTTCAGCAAAGG
**Acta2**	Forward		CCCAGACATCAGGGAGTAATGG
	Reverse		TCTATCGGATACTTCAGCGTCA
**MMP2**	Forward	CCCCAAAACGGACAAAGAG	
	Reverse	CTTCAGCACAAACAGGTTGC	
**IL-1α**	Forward	TGGTAGTAGCAACCAACGGGA	CGAAGACTACAGTTCTGCCATT
	Reverse	ACTTTGATTGAGGGCGTCATTC	GACGTTTCAGAGGTTCTCAGAG
**IL-1β**	Forward	CTCCAGGGACAGGATATGGA	TGTGTAATGAAAGACGGCACA
	Reverse	TCTTTCAACACGCAGGACAG	GGGGAACTCTGCAGACTCAA
**IL-6**	Forward	ACTCACCTCTTCAGAACGAATTG	TAGTCCTTCCTACCCCAATTTCC
	Reverse	CCATCTTTGGAAGGTTCAGGTTG	TTGGTCCTTAGCCACTCCTTC
**IL-8**	Forward	TTGGCAGCCTTCCTGATTTC	
	Reverse	TATGCACTGACATCTAAGTTCTTTAGCA	
**IL-10**	Forward	GACTTTAAGGGTTACCTGGGTTG	
	Reverse	TCACATGCGCCTTGATGTCTG	
**IL-23a**	Forward	CTCAGGGACAACAGTCAGTTC	
	Reverse	ACAGGGCTATCAGGGAGCA	
**TNF-α**	Forward	GAGGCCAAGCCCTGGTATG	GATTATGGCTCAGGGTCCAACTCT
	Reverse	CGGGCCGATTGATCTCAGC	GGACATTCGAGGCTCCAGTGAATT
**CCL3**	Forward	AGTTCTCTGCATCACTTGCTG	
	Reverse	CGGCTTCGCTTGGTTAGGAA	
**CCL5**	Forward	CCAGCAGTCGTCTTTGTCAC	
	Reverse	CTCTGGGTTGGCACACACTT	
**CCL20**	Forward	TGCTGTACCAAGAGTTTGCTC	
	Reverse	CGCACACAGACAACTTTTTCTTT	
**HB-EGF**	Forward	ATCGTGGGGCTTCTCATGTTT	
	Reverse	TTAGTCATGCCCAACTTCACTTT	
**FGF1**	Forward	CTCCCGAAGGATTAAACGACG	

	Reverse	GTCAGTGCTGCCTGAATGCT
**FGF2**	Forward	AGAAGAGCGACCCTCACATCA
	Reverse	CGGTTAGCACACACTCCTTTG
**FGF5**	Forward	CACTGATAGGAACCCTAGAGGC
	Reverse	CAGATGGAAACCGATGCCC
**FGF7**	Forward	TCCTGCCAACTTTGCTCTACA
	Reverse	CAGGGCTGGAACAGTTCACAT
**IGF-1**	Forward	GCTCTTCAGTTCGTGTGTGGA
	Reverse	GCCTCCTTAGATCACAGCTCC
**IGF-2**	Forward	GTGGCATCGTTGAGGAGTG
	Reverse	CACGTCCCTCTCGGACTTG
**TNFR1**	Forward	TCACCGCTTCAGAAAACCACC
	Reverse	GGTCCACTGTGCAAGAAGAGA
**TNFR2**	Forward	CGGGCCAACATGCAAAAGTC
	Reverse	CAGATGCGGTTCTGTTCCC

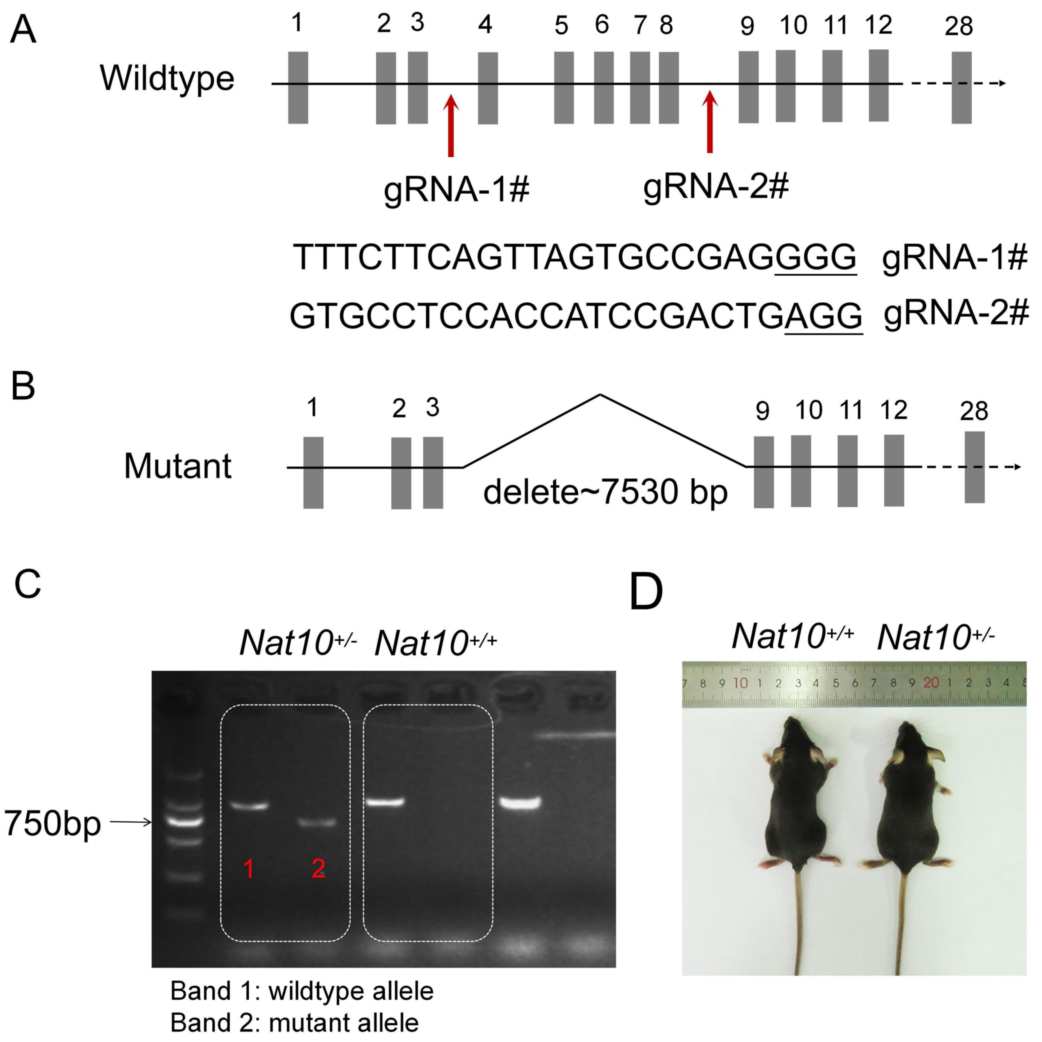

Fig.S1 Generation of *Nat10* knockout mice

(A) The diagram showing the design of gRNA-1 and gRNA-2 targeting *Nat10* genome.

(B) The diagram showing the deletion of *Nat10* genome between exon 4 and exon 8.

(C) *Nat10*$^{+/-}$ and WT mice were verified by allele-specific PCR genotyping.

(D) The photo displayed the morphology comparison between WT and *Nat10*$^{+/-}$ mice at the age of 2-month-old.

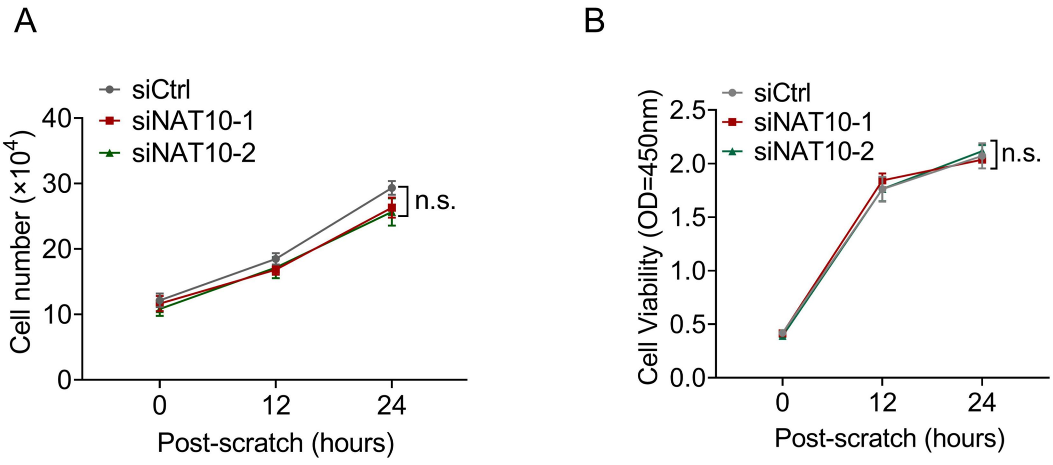

Fig.S2 NAT10 knockdown affects little on cell proliferation and viability during in vitro wound healing

(A) Curves showing proliferation of HaCaT cells with or without NAT10 knockdown in the scratch-wounded cell model.

(B) Cell viability was detected by CCK8 assays in HaCaT cells with treatment of NAT10 knock down or not.

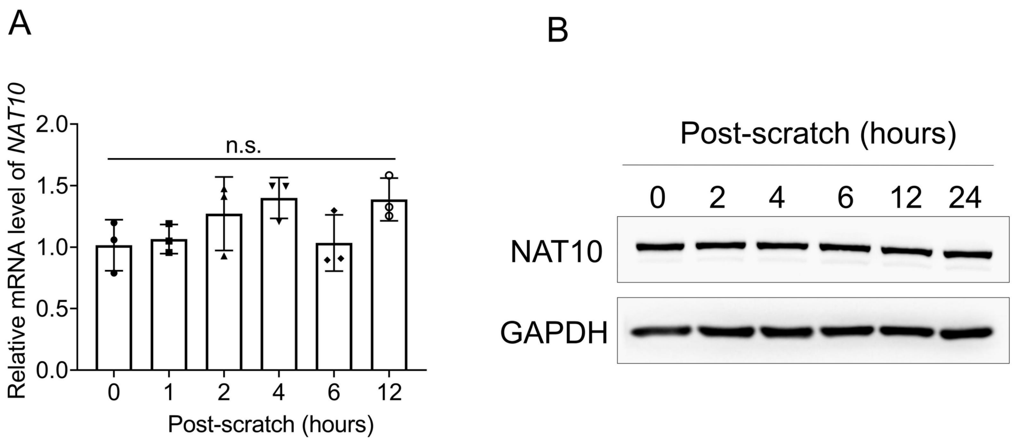

Fig.S3 NAT10 expression is not significantly changed in keratinocyte cells during wound healing process

(A) qPCR analysis of *NAT10* mRNA level in the scratch-wounded HaCaT cells.

(B) Immunoblot analysis of NAT10 in HaCaT cells post the scratch-induced wound.

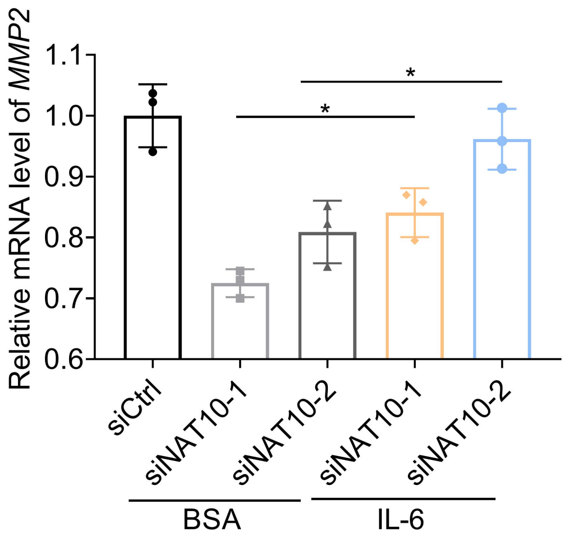

Fig.S4 rIL6 supplementation restores MMP2 expression in NAT10 knocked down HaCaT cells

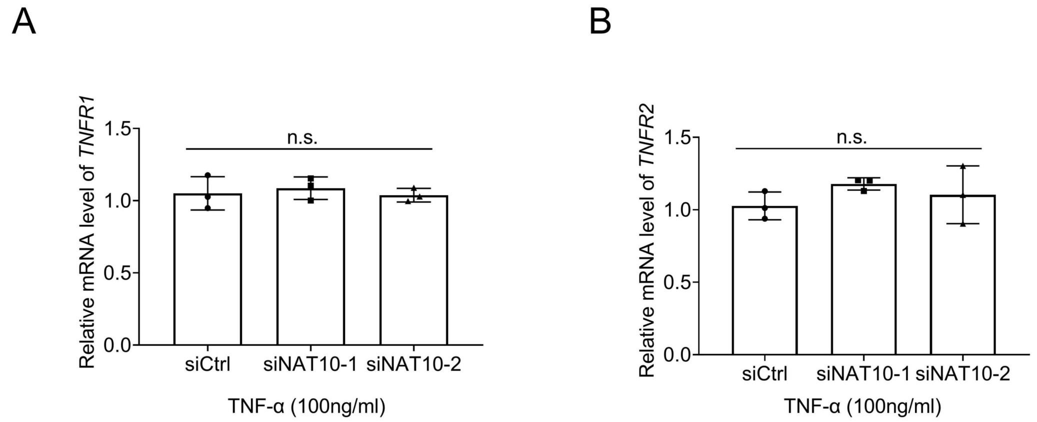

Fig.S5 NAT10 knockdown has less effect on TNFR1 or TNFR2 expression in HaCaT cells

(A) Quantifying relative levels of TNFR1/GAPDH in HaCaT cells with or without NAT10 knockdown and TNFα treatment from 3 independent experiments.

(B) Quantifying relative levels of TNFR2/GAPDH in HaCaT cells with or without NAT10 knockdown and TNFα treatment from 3 independent experiments.

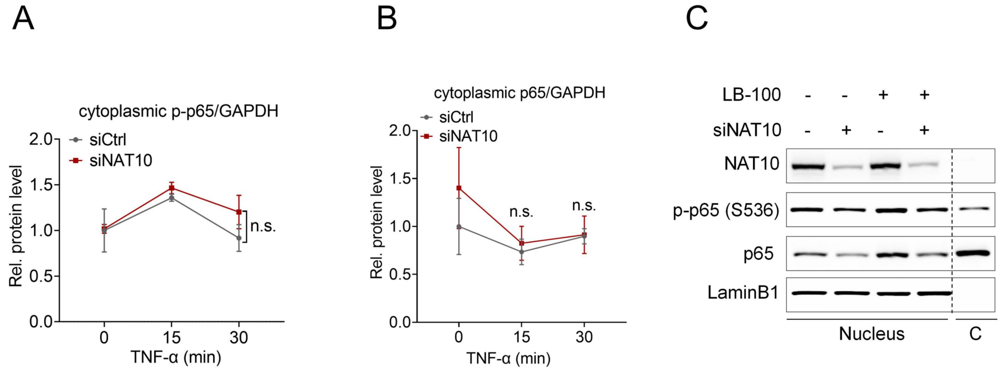

Fig.S6 NAT10 does not affect cytoplasmic p65 activation and PP2A mediate dephosphorylation

(A and B) Quantifying relative levels of cytoplasmic p-p65/GAPDH and p65/GAPDH in HaCaT cells with or without NAT10 knockdown and TNFα treatment from 3 independent experiments.

(C) Representative immunoblotting showing the nuclear p65 expression in LB-100-treated HaCaT cells with controlled or NAT10 siRNA transfection.

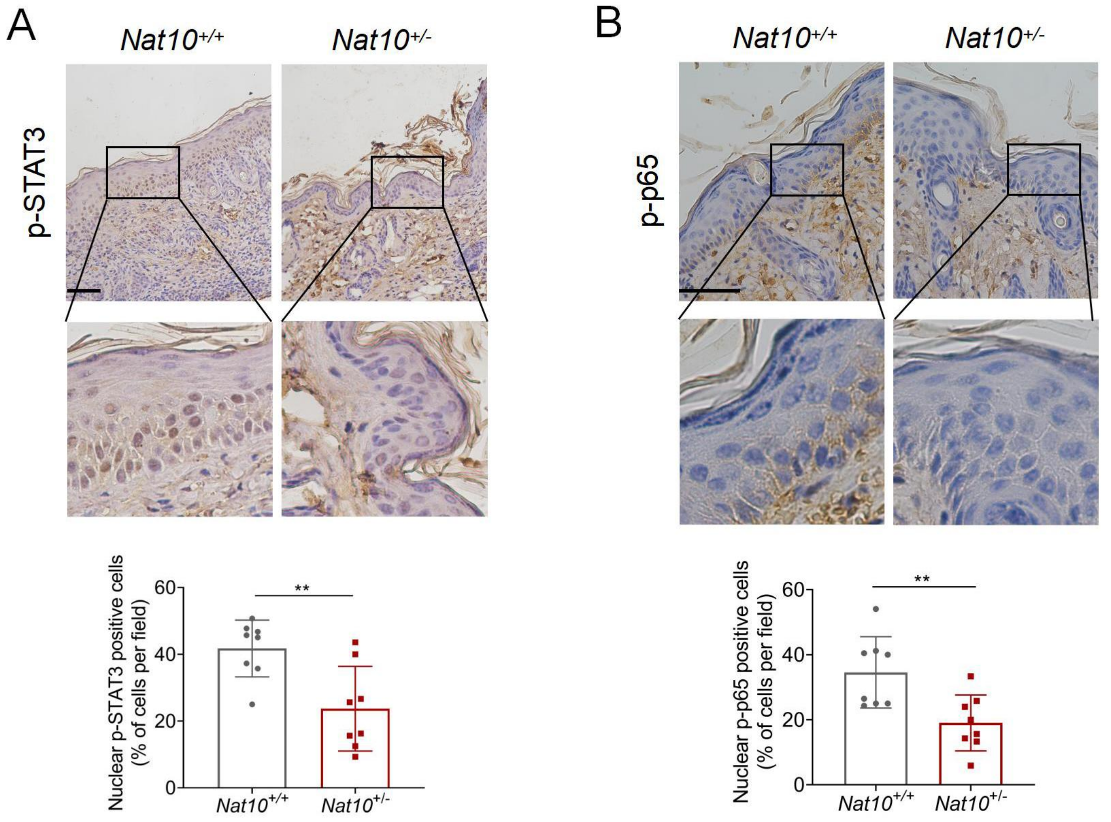

Fig.S7 IHC staining analysis of p-p65 and p-stat3 in the wound skin tissues

(A) The IHC staining of p-STAT3 in the wound skin tissues derived from WT and *Nat10*$^{+/-}$ mice on day 5 after punching. All quantifications were restricted to the epidermis. Scale bar, 20 μm.

(B) The IHC staining of p-p65 (Phospho-NF-κB p65 (Ser536)) in the wound skin tissues derived from WT and *Nat10*$^{+/-}$ mice on day 5 after punching. All quantifications were restricted to the epidermis. Scale bar, 20 μm.
