# Supplementary material for: N-acetyltransferase 10 promotes cutaneous wound repair via the NF-κB-IL-6 axis
Source: Cell Death Discov. 2023 Aug 29;9:324. doi: 10.1038/s41420-023-01628-2 (PMC10465497; doi:10.1038/s41420-023-01628-2)

Figure 1G

NAT10

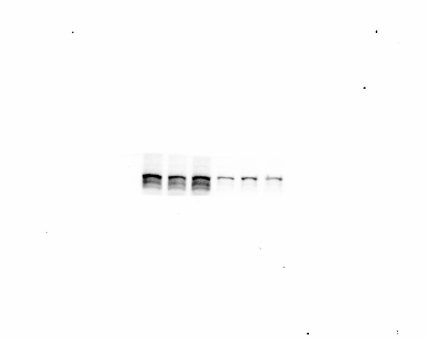

aSMA

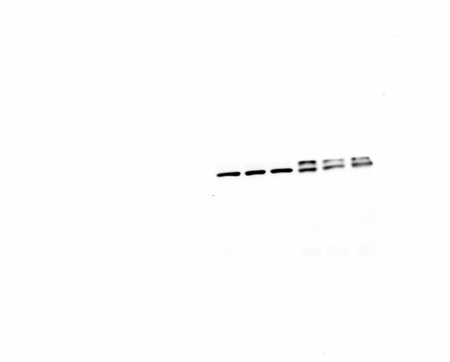

FN1

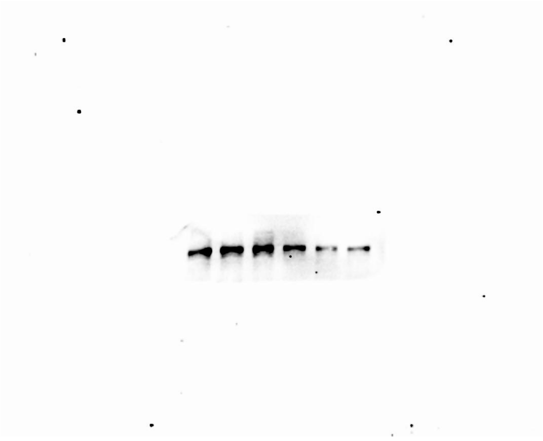

tubulin

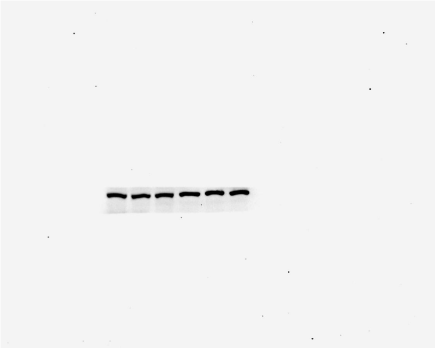

Figure 2B

NAT10 (right 3 lanes)

Tubulin (right 3 lanes)

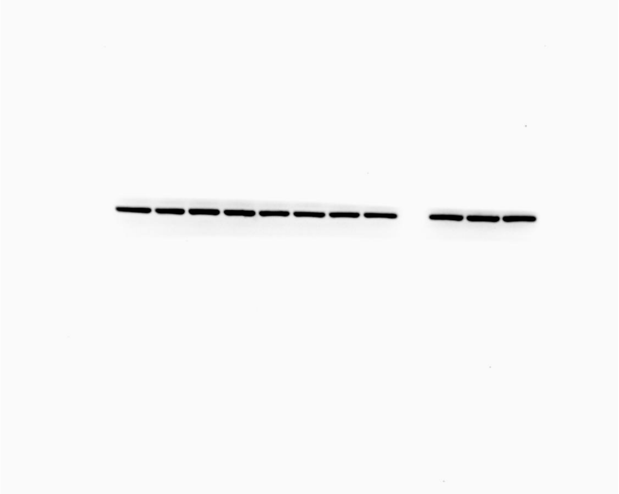

Figure 2I

NAT10

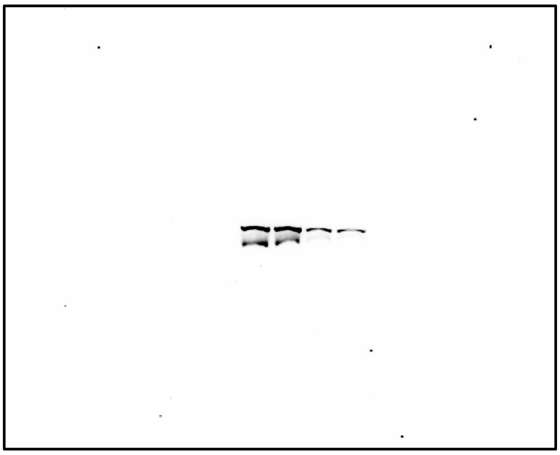

P-FAK

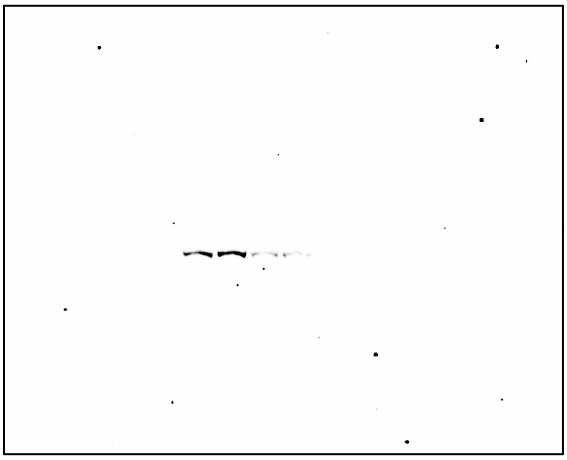

T-FAK

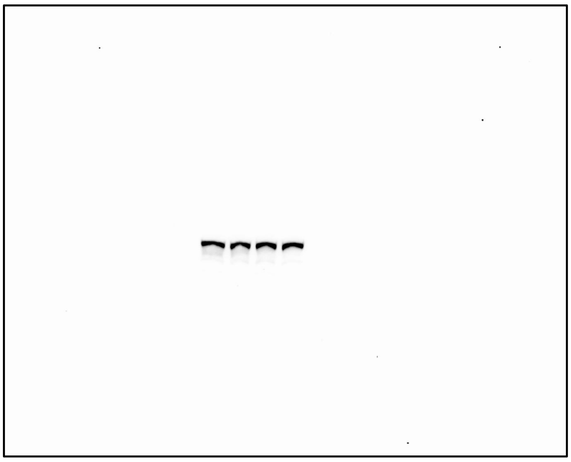

Tubulin

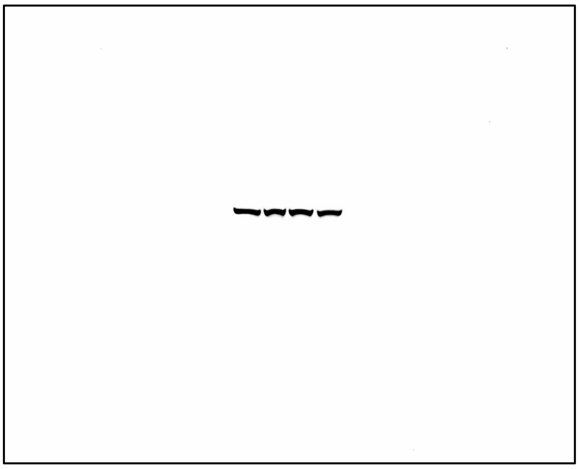

Figure 3D

p-stat3 left 4 lanes

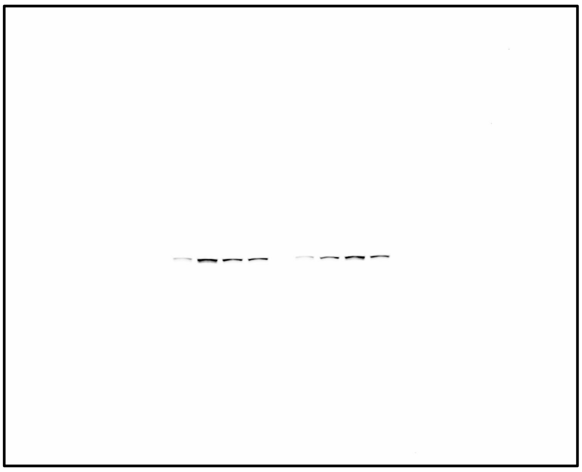

T-stat3

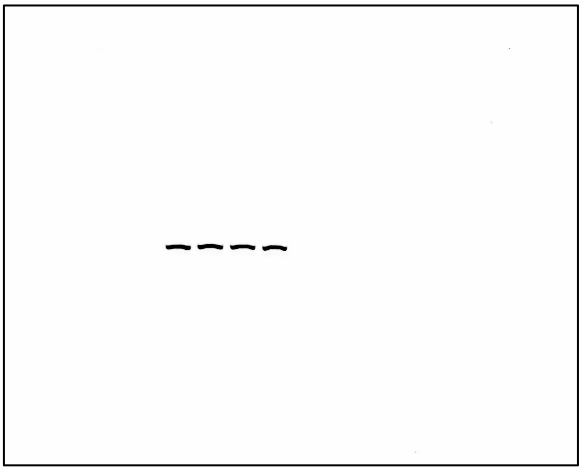

Tubulin upper-left 4 lanes

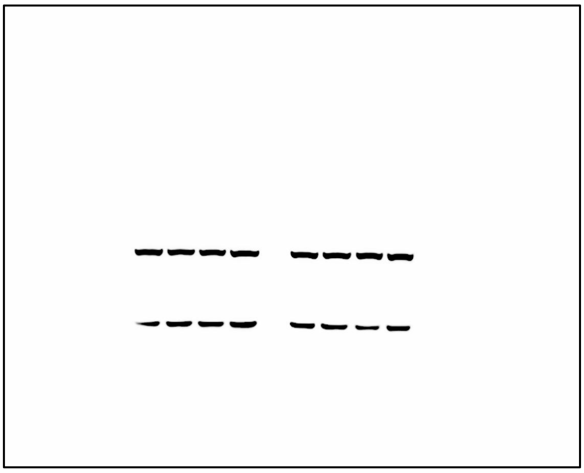

Figure 3I

T-stat3

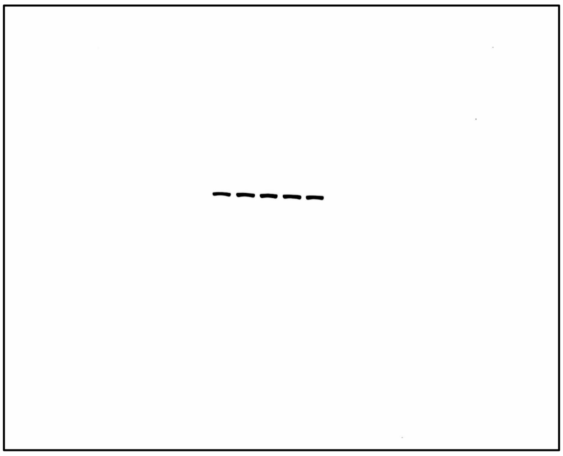

P-stat3

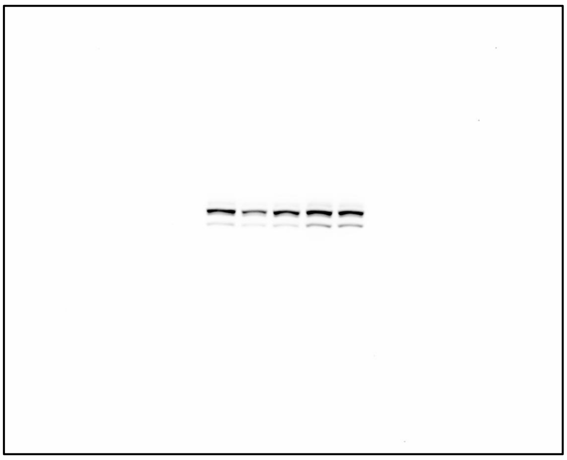

NAT10

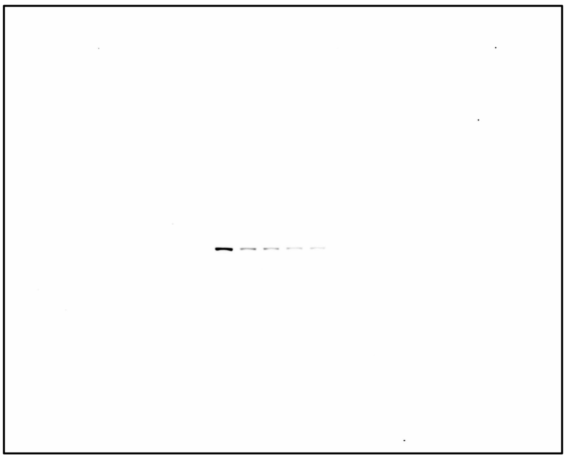

Tubulin (upper)

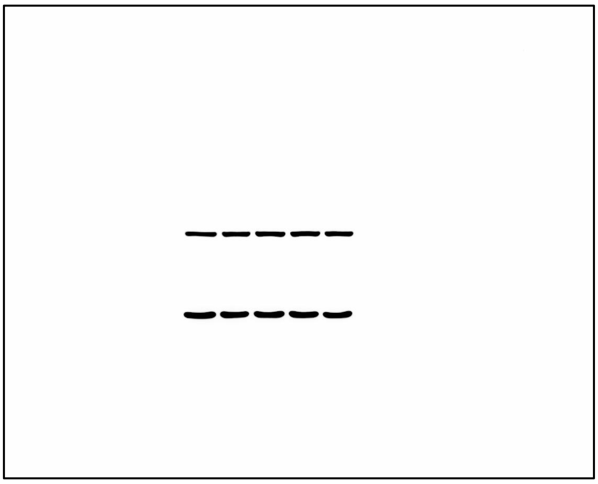

Figure 4A

NAT10

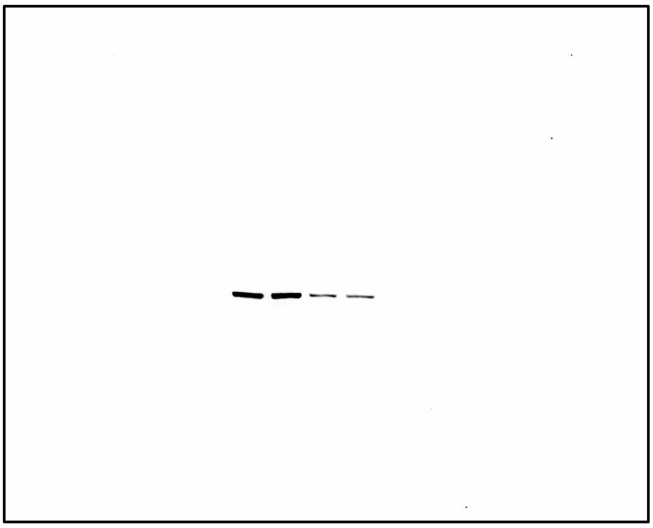

T-p65

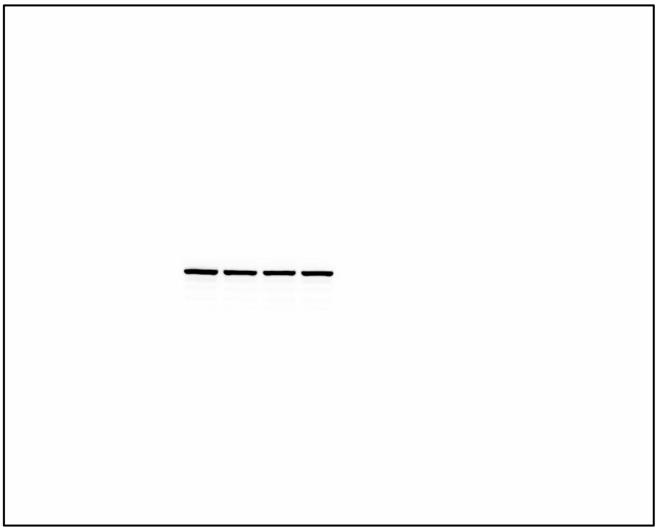

P-p65

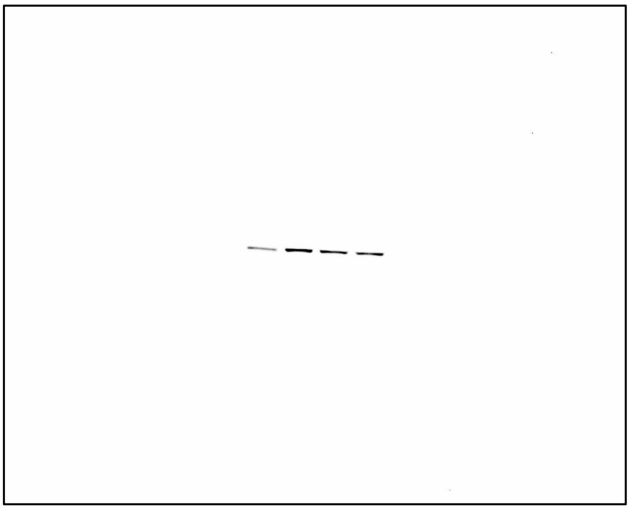

Tubulin (below)

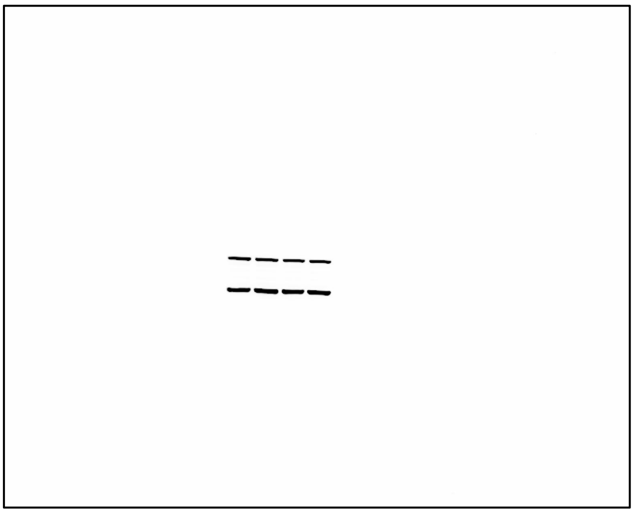

Figure 4E

IKKa (upper-left 4 lanes)

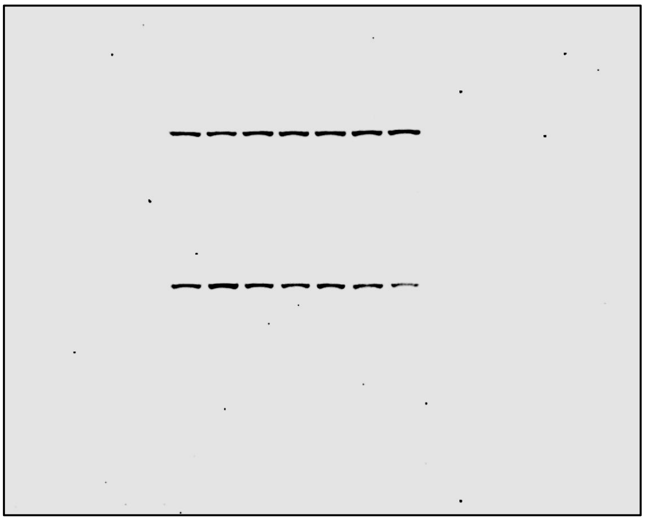

T-p65 (left 4 lanes)

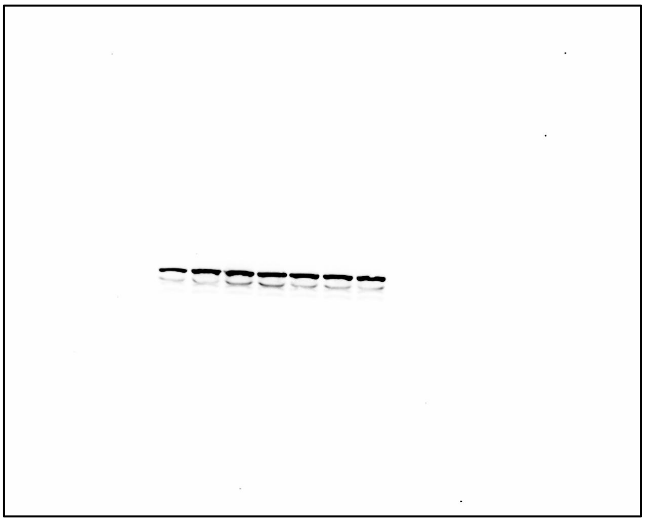

p-p65 (left 4 lanes)

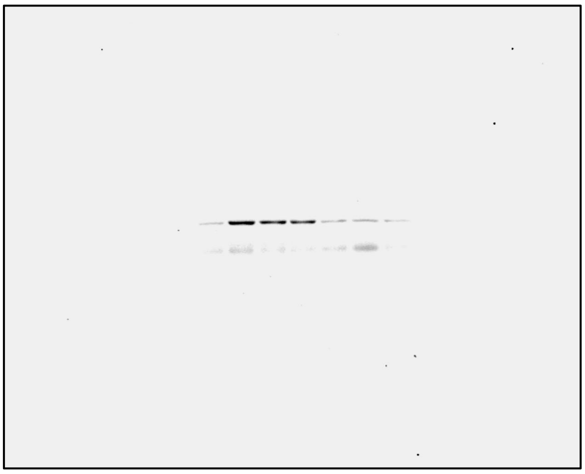

Ikkb (left 4 lanes)

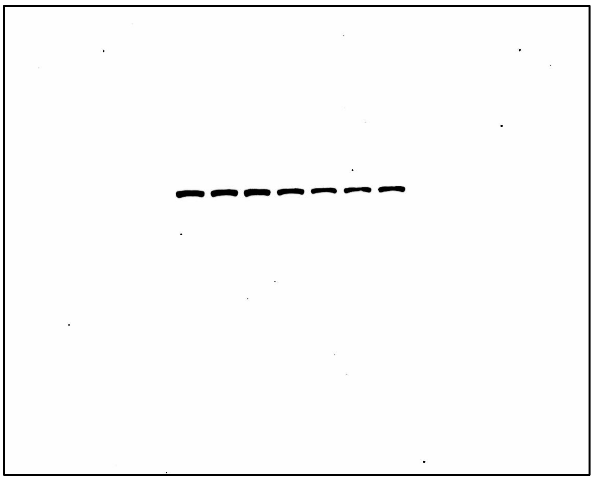

TNF nat10 (left 4 lanes)

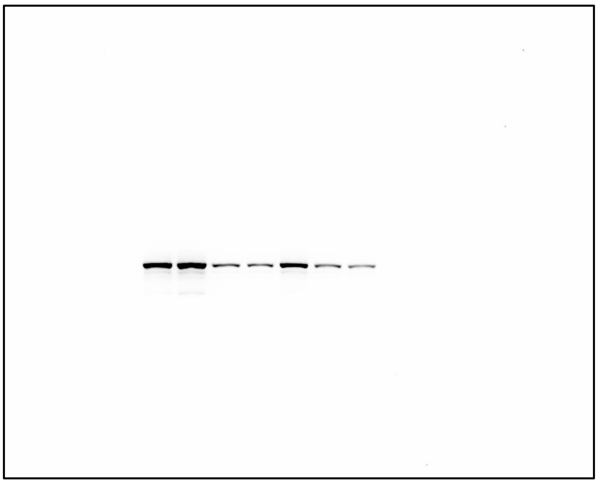

TNF pikkab (left 4 lanes)

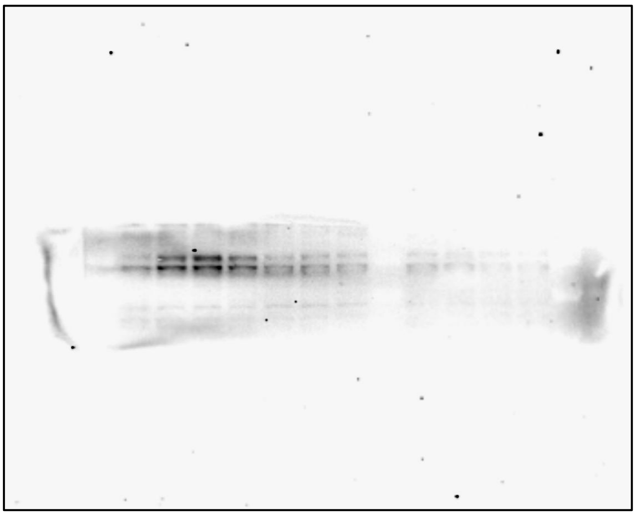

Figure 5A

Cyto t-p65

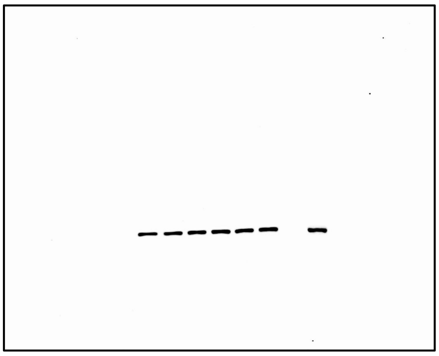

Cyto p-p65

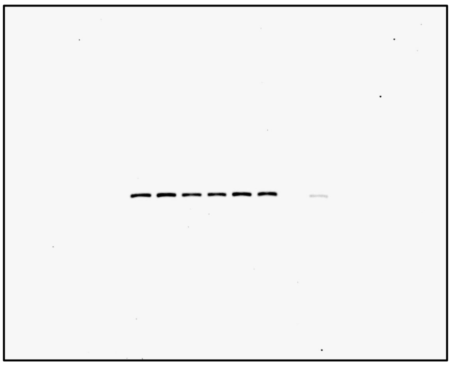

Cyto nat10

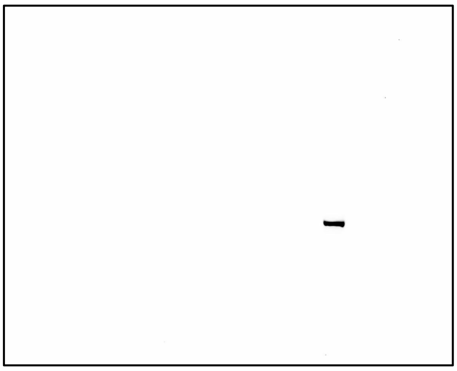

Cyto laminB1 (upper)

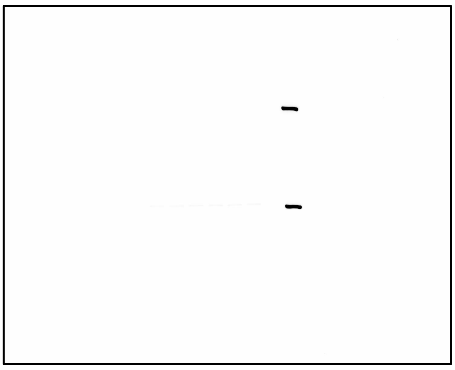

Cyto GAPDH (upper)

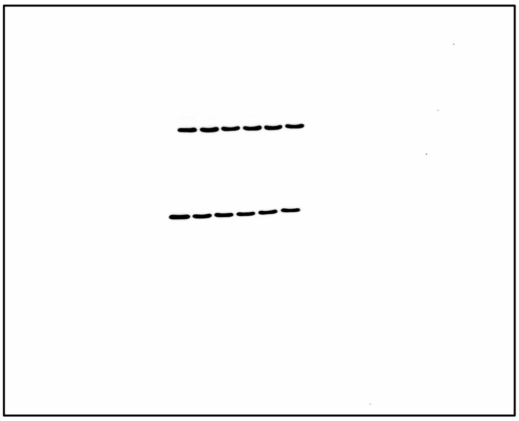

nuclear GAPDH (upper)

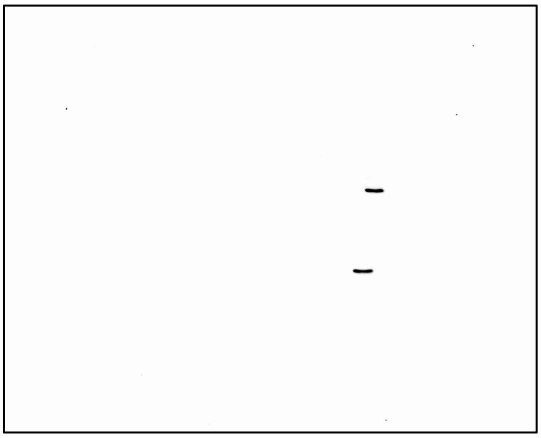

nuclear laminB (upper)

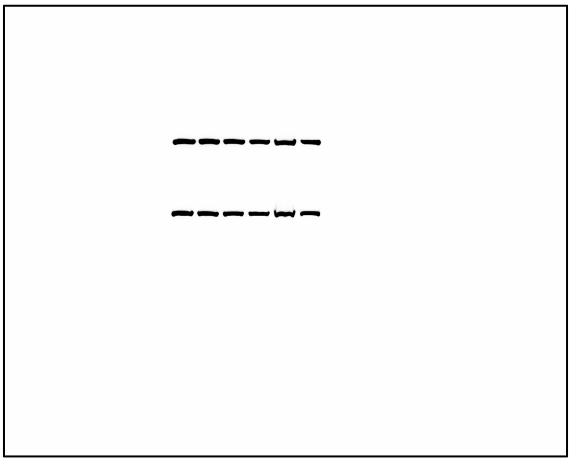

nuclear nat10 (upper)

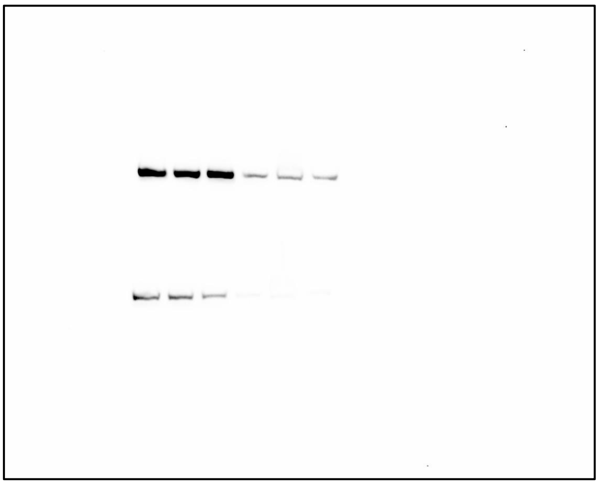

nuclear t-p65

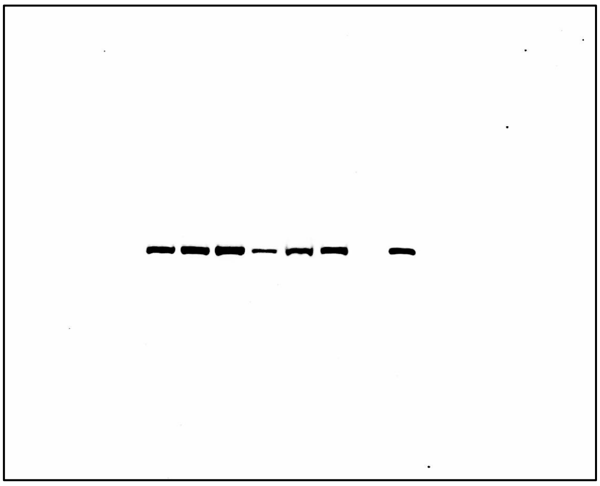

nuclear p-p65

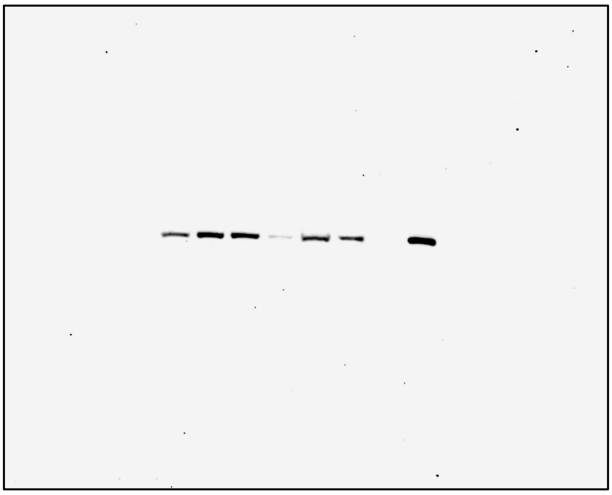

Figure 5D

Nuclear lamin B1 (upper 0/1/2/4 hours)

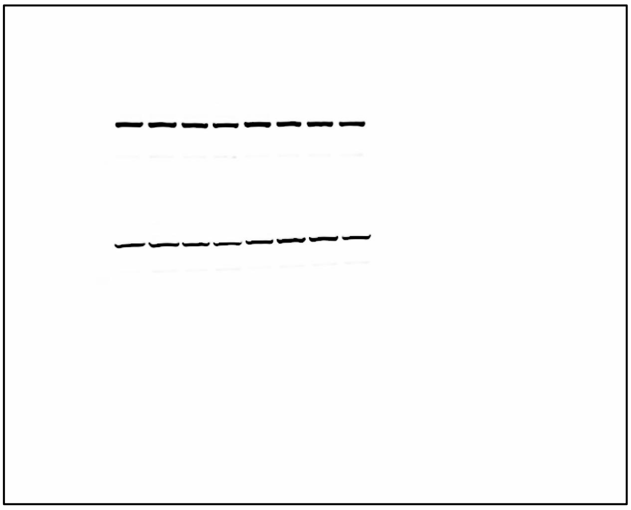

Nuclear t-p65 (upper 0/1/2/4 hours)

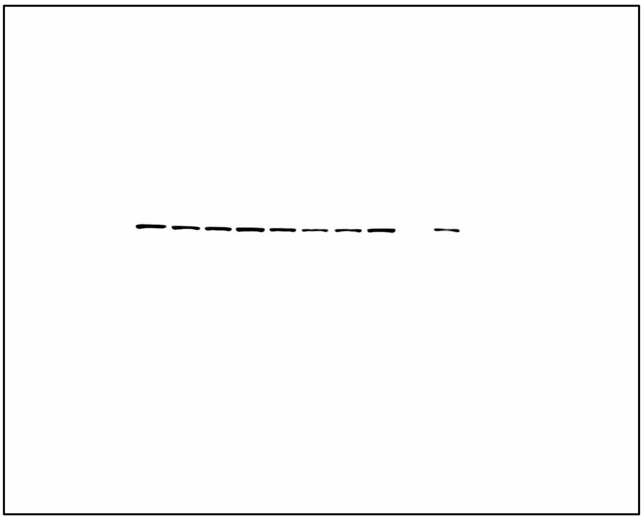

Figure 5F

input laminB1 (below-right 2 lanes)

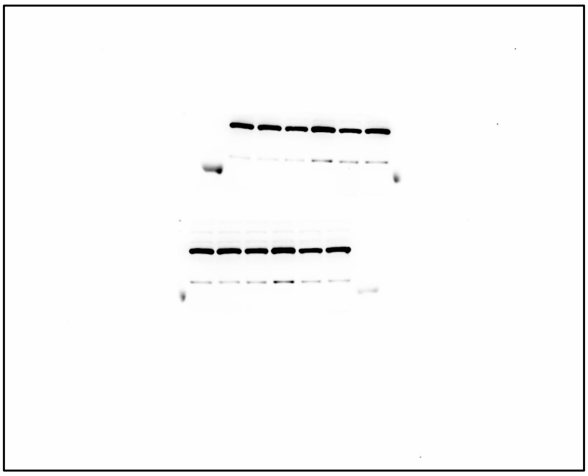

input nat10 (right 2 lanes)

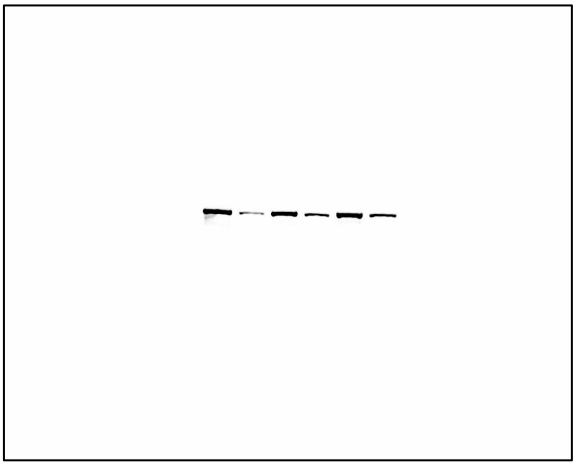

ip p65 upper (right 2 lanes)

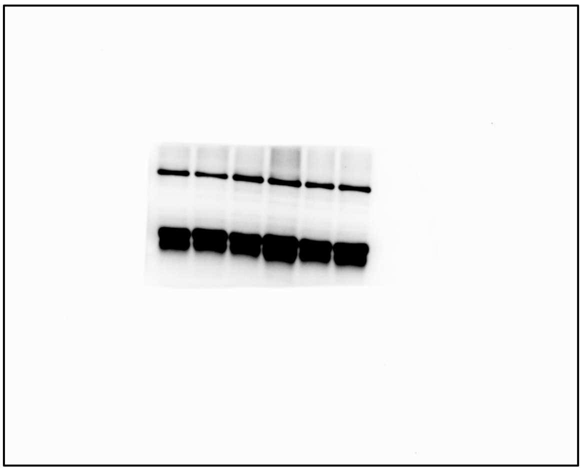

UB (right 2 lanes)

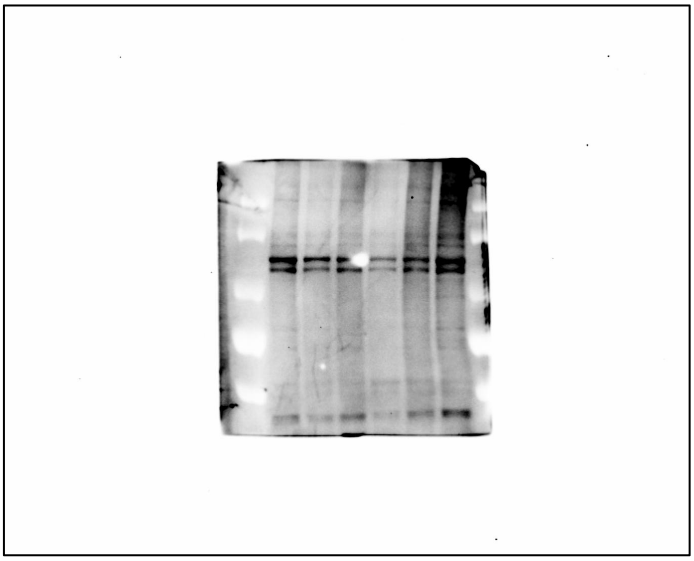

Figure 6A

Mice p-p65

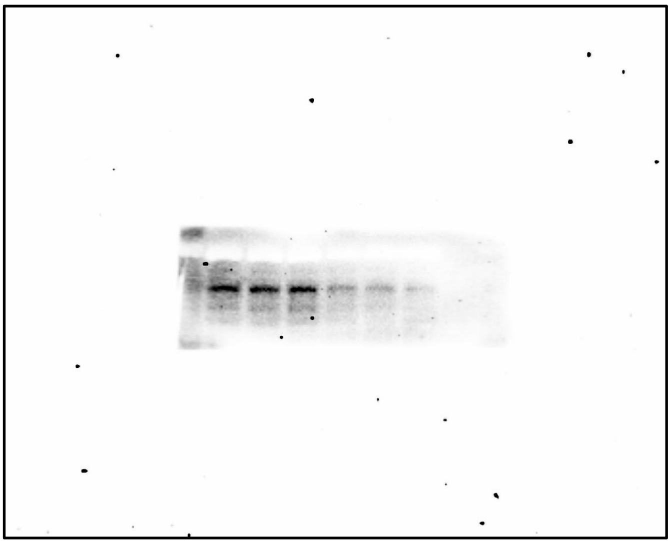

Mice t-p65

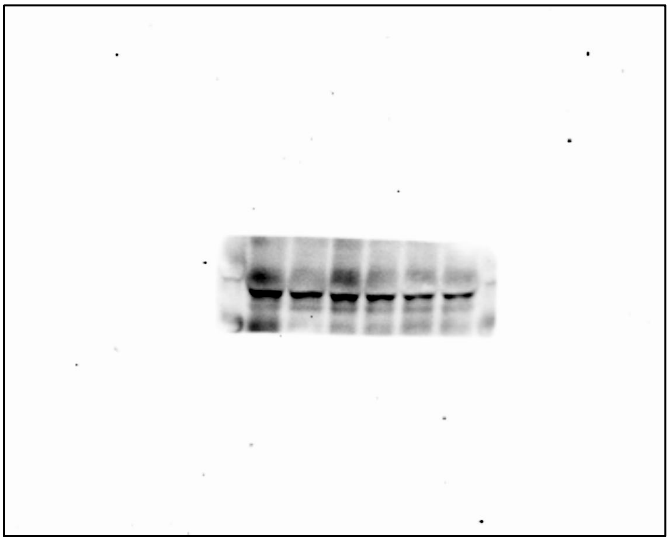

Mice p-stat3

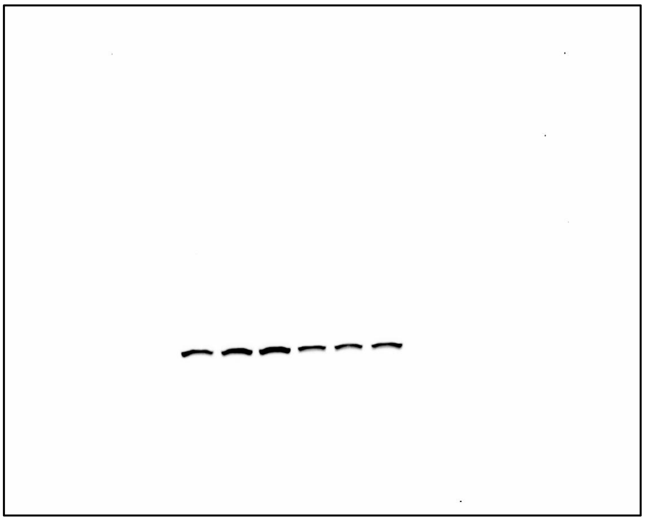

Mice t-stat3

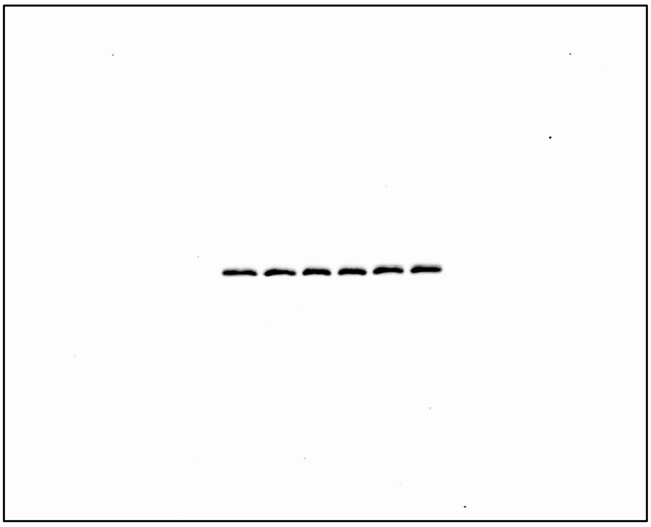

Supplement: Supplementary file 2 — Original Data File [file 41420_2023_1628_MOESM2_ESM.pdf]
